# Small secreted peptides (SSPs) in tomato and their potential roles in drought stress response

**DOI:** 10.1186/s43897-023-00063-2

**Published:** 2023-08-25

**Authors:** Kexin Xu, Dongdong Tian, TingJin Wang, Aijun Zhang, Mohamed Abdou Youssef Elsadek, Weihong Liu, Liping Chen, Yongfeng Guo

**Affiliations:** 1 Department of HorticultureCollege of Agriculture and Biotechnology, Zhejiang University, Hangzhou, 310058 China; 2 Tobacco Research Institute, Chinese Academy of Agricultural Sciences, Qingdao, 266101 China

**Keywords:** Small secreted peptide, Tomato, Drought stress, CEP peptide

## Abstract

Tomato (*Solanum lycopersicum*) is one of the most important vegetable crops in the world and abiotic stresses often cause serious problems in tomato production. It is thus important to identify new regulators in stress response and to devise new approaches to promote stress tolerance in tomato. Previous studies have shown that small secreted peptides (SSPs) are important signal molecules regulating plant growth and stress response by mediating intercellular communication. However, little is known about tomato SSPs, especially their roles in responding to abiotic stresses. Here we report the identification of 1,050 putative SSPs in the tomato genome, 557 of which were classified into 38 known SSP families based on their conserved domains. GO and transcriptome analyses revealed that a large proportion of *SlSSPs* might be involved in abiotic stress response. Further analysis indicated that stress response related cis-elements were present on the *SlCEP* promotors and a number of *SlCEPs* were significantly upregulated by drought treatments. Among the drought-inducible *SlCEP*s, SlCEP10 and SlCEP11b were selected for further analysis via exogenous application of synthetic peptides. The results showed that treatments with both SlCEP10 and SlCEP11b peptides enhanced tomato drought stress tolerance, indicating the potential roles of SlSSPs in abiotic stress response.

**Graphical Abstract:**

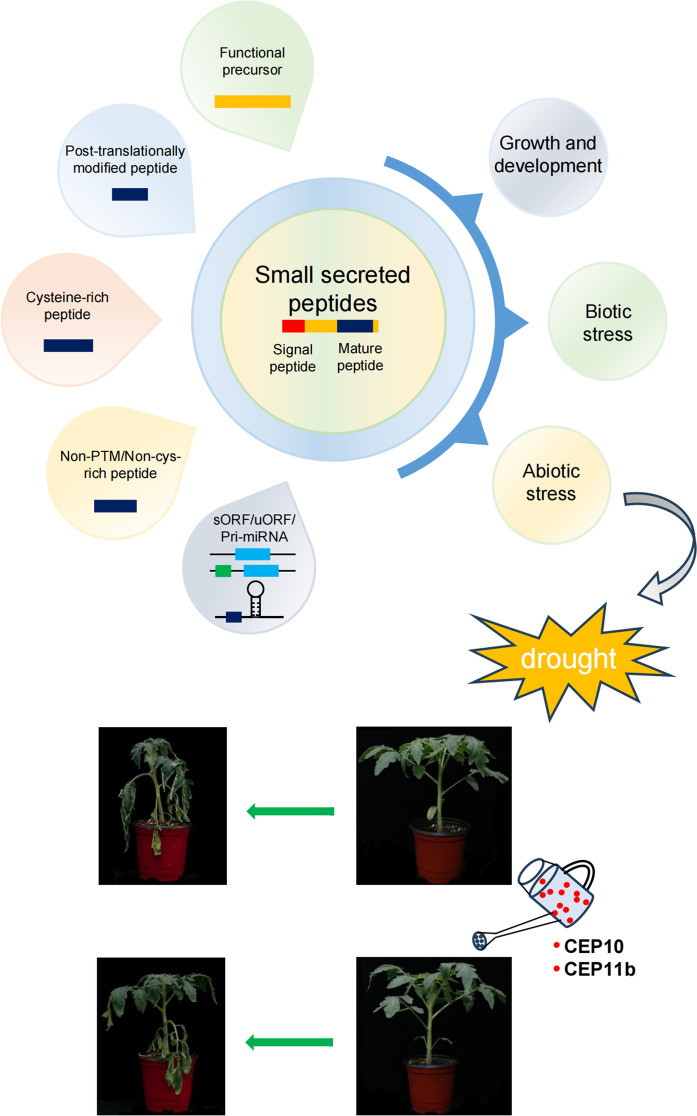

**Supplementary Information:**

The online version contains supplementary material available at 10.1186/s43897-023-00063-2.

## Core

One thousand fifty putative SSPs in tomato were identified and classified using a multi-step procedure. The SlCEP10 and SlCEP11b peptides were found to be able to enhance the tolerance of tomato plants to drought stress when exogenously applied.

## Gene & accession numbers

A list of genes and sequences used in the qRT-PCR analysis can be found in Supplementary Table S4.

## Introduction

Small secreted peptides (SSPs) are signal molecules that play key roles in plant growth, development and stress response by mediating cell to cell communication (Vie et al. 2015; Takahashi et al. 2018). Since the identification of systemin, a plant signaling peptide involved in defense response of tomato leaves in early nineties (Pearce et al. 1991), an increasing number of SSPs were identified in a variety of plant species in the past 30 years. Plant SSPs have been shown to be involved in diverse processes of plant growth and development, including cell division and differentiation within meristems (Fletcher et al. 1999; Pallakies and Simon 2014), cellular longevity and plant senescence (Matsubayashi et al. 2006; Zhang et al. 2022), root growth and nutrient availability (Taleski et al. 2018; Chapman et al. 2019), floral organ abscission (Reichardt et al. 2020), stomatal density and distribution (Lee et al. 2015) and stress response (Nakaminami et al. 2018; Takahashi et al. 2018; Aggarwal et al. 2020; Liu et al. 2022).

SSP signals are derived from precursor proteins that share the following characteristics: short length, usually less than 250 amino acids; with an N-terminal signal peptide for secretory pathway and a C-terminal conserved mature peptide (Lease and Walker 2006; Marshall et al. 2011). Based on the function of SSP precursors, SSPs can be divided into two major groups: functional precursors and SSP with unfunctional precursors. The latter can be further categorized into three classes based on their mature form: post-translationally modified (PTM) peptides, Cys-rich peptides (CRP), and Non-Cys-rich/Non-PTM peptides. PTMs contain small size mature peptides with post-translational modifications (Matsubayashi 2011), including PAMP-induced peptides (PIPs), C-terminally encoded peptides (CEPs), CLAVATA3 (CLV3)/ESR peptides (CLEs), inflorescence deficient in abscission (IDA), phytosulfokine (PSK) and plant peptide containing sulfated tyrosine (PSY) etc. (Matsubayashi and Sakagami 1996; Fletcher et al. 1999; Butenko et al. 2003; Sawa et al. 2006; Amano et al. 2007; Ohkubo et al. 2017; Zhou et al. 2022). CRPs contain an even number of cysteine residues that are necessary for the formation of intramolecular disulfide bonds, including rapid alkalinization factors (RALFs), STOMAGEN and epidermal patterning factors (EPFs) etc. (Pearce et al. 2001; Hara et al. 2007; Sugano et al. 2010). Non-Cys-rich/non-PTM peptides include systemin (SYS) and plant elicitor peptides (PEPs) etc. (Pearce et al. 1991; Nakaminami et al. 2018). With more advanced techniques in identifying small secreted peptides becoming available, more and more peptide families have been identified and characterized.

Based on the basic structure of SSPs, bioinformatic approaches have been applied to uncover new members of known gene families and new peptide families from genomic sequences of various plant species. Lease and Walker wrote a Perl script to identify unannotated *Arabidopsis* peptides and reported 33,809 putative ORFs encoding SSPs (Lease and Walker 2006). Hanada et al. used the ‘Coding Index’ method in identifying 7,159 coding sORFs with length between 30 and 100 aa in *Arabidopsis*, with a claimed 1% false discovery rate (Hanada et al. 2007; Hanada et al. 2010). Pan et al. obtained 101,048 SSP candidates in rice by screening the whole genome through six-frame translation in EMBOSS and gene modeling through Augustus and FGENESH (Pan et al. 2013). Li et al. identified 1,491 putative SSPs from the maize genome (Li et al. 2014). Boschiero et al. identified 4,439 SSPs in *M. truncatula* via a multistep analytical procedure (Boschiero et al. 2020). Wang et al. used a combined transcriptomics- and proteomics-based screenings and isolated 236 SSP candidates involved in rice immunity (Wang et al. 2020). Tian et al. identified 4,981 putative wheat SSPs with protein length less than 250 aa (Tian et al. 2022). Certain SSP families such as CLEs (Carbonnel et al. 2022) and CEPs (Liu et al. 2022) have been characterized in more details for their functions in plant development and stress response.

Plants, as sessile organisms, are exposed to various environmental stresses during their lifecycle. To cope with unfavorable environments, plants have developed complex physiological and molecular defense mechanisms to sense and adapt to stresses. Recently, the roles of small secreted peptides in plant stress response have become the focus of a number of studies (Chen et al. 2020a, b). In *Arabidopsis* CLE25 and CLE9 peptides were characterized to control stomatal closure and prevent water loss under dehydration through ABA signaling (Takahashi et al. 2018; Zhang et al. 2019). The PEP3 peptide is recognized by the PEPR1 receptor and plays a significant role in salinity stress tolerance (Nakaminami et al. 2018). RALF peptides (RALF1/RALF22/23) regulate salinity response via distinct mechanisms (Feng et al. 2018; Yu and Assmann 2018; Zhao et al. 2021). *CEP5* expression is highly induced by osmotic stress and synthetic peptide treatments/overexpression of *CEP5* enhanced the tolerance to drought stress (Smith et al. 2020). PIP3 plays an essential role in plant salt tolerance through binding to the RLK7 receptor and activating the MPK3/MPK6 cascade (Zhou et al. 2022). PSK triggers premature flower drop in tomato under drought stress, which is an indispensable regulated process for plant development (Reichardt et al. 2020).

As the most important horticultural crop worldwide, tomato (*Solanum lycopersicum*) is also a model plant for physiological and molecular research in addition to *Arabidopsis* (Lin et al. 2014). Drought, salt, cold and combined abiotic and biotic stresses often cause serious problems in tomato production (Bai et al. 2018). Among them drought is the most serious stress condition, which inhibits plant growth and affects flowering and fruit setting in tomato, (Chong et al. 2022). So far, no comprehensive identification and classification of SSPs from the tomato genome have been reported. Here, we report the identification of 1,050 putative SSPs from the tomato genome and analysis of the expression patterns of representative *SSP* genes in different tissues and under drought stress conditions. Two tomato CEP peptides, SlCEP10 and SlCEP11b, were further characterized for their role in improving drought tolerance in tomato plants through exogenous peptide application. These findings suggested that SlSSPs might play an important role in tomato drought stress response.

## Results

### Identification of SSPs in tomato

**Fig. 1:**
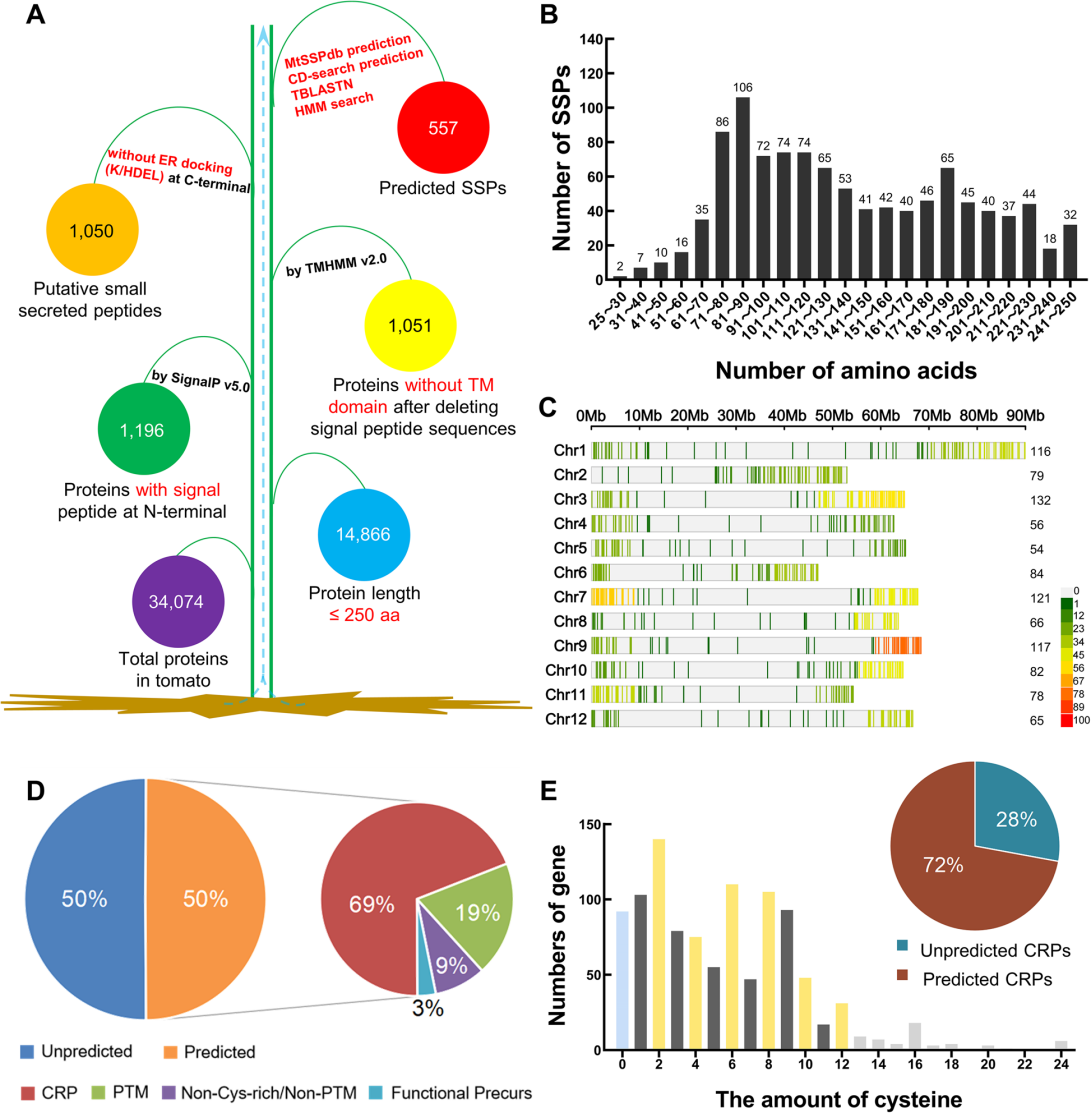
Identification and classification of SSPs in tomato. **A** The procedure of tomato SSP identification. **B** The number of SlSSPs with different protein length ranges. **C** Chromosomal distribution of *SlSSPs*. *SlSSP* density plot on each chromosome represented by number of *SSPs* within 1 Mb window size. Chromosome distribution visualization was created by an online platform (https://www.bioinformatics.com.cn). **D** The percentage of different types of SlSSPs predicted in MtSSPdb (https://mtsspdb.noble.org/database/). CRP, cysteine-rich peptide; PTM, post-translational modified. **E** The prediction of CRPs in tomato. The column represents amount of SlSSPs with different number of cysteines after deleting signal peptide sequences, the pie represents the percentage of predicted CRPs and unpredicted CRPs in MtSSPdb

In order to identify SSPs in tomato, all the predicted protein sequences of tomato were downloaded from Phytozome (https://phytozome-next.jgi.doe.gov/) and SGN (https://solgenomics.net/). Firstly, we obtained 14,866 small proteins with no more than 250 amino acid residues. Next 1,051 putative small peptide proteins were obtained by removing proteins lacking an N-terminal signal peptide and proteins with transmembrane domains, which were predicted using SignalP-5.0 (https://services.healthtech.dtu.dk/service.php?SignalP) (Almagro Armenteros et al. 2019) and TMHMM v2.0 (https://services.healthtech.dtu.dk/service.php?TMHMM-2.0) (Krogh et al. 2001) respectively. Finally, a total of 1,050 putative tomato SSPs (SlSSPs) were identified after removing a putative endoplasmic reticulum docking protein based on the presence of a C-termini HDEL domain (Fig. 1A, Supplementary Table S1).

The length of most of the SlSSPs ranges from 61 to 230 amino acid residues, with only 35 SSPs (3%) being shorter than or equal to 60 residues (Fig. 1B). The molecular weight of the SlSSPs ranges from 2.66 kDa to 27.97 kDa, and the isoelectric point ranges from 3.22 to 12.71 (Supplementary Table S1). The results of chromosome localization analysis indicate that the 1,050 SlSSP-encoding genes are evenly distributed on the chromosomes, except for Chr4 and Chr5, which contains only 56 and 54 *SSP* genes respectively. Interestingly, most of the *SlSSP* genes are located at the ends of each chromosome. For example, both ends of Chr9 are enriched with *SlSSP* genes (Fig. 1C).

### Classification of SSPs in tomato

The *Medicago truncatula* Small Secreted Peptide Database (MtSSPdb; http://mtsspdb.noble.org/database/) and Web CD-search Tool (https://www.ncbi.nlm.nih.gov/Structure/bwrpsb/bwrpsb.cgi) were used to perform protein family classification of the SlSSPs. The results of classification were further confirmed using the HLH hidden Markov model (HMM) search, homologue BLAST and manual sequence alignment to ensure accuracy and completeness. In total, 557 putative SlSSPs were grouped into 38 known SSP families, belonging to four classes: Post-translationally modified (PTM, 19%), Cysteine rich (CRP, 69%), Non-Cys-rich/Non-PTM (9%) and Functional Precursor (3%) (Fig. 1D, Table 1). Based on predicted functions, the 557 SlSSPs with known peptide domains can be classified as signal peptides (72%), antimicrobial peptides (15%), peptidase inhibitors (11%) and unknown peptides (2%) (Table 1). Among the 1,050 SlSSPs, 534 (50.86%) proteins contain even number (2–16) of cysteine residues at their C-terminal and were considered as putative CRPs (Fig. 1E). Only 72% of the putative CRPs were confirmed in MtSSPdb, indicating that there are still some novel SSPs in tomato that need to be characterized.

**Table 1:** Predicted SSP families in tomato

Class	SSP family	Description	Mode of action	Number of peptides
Post-translationally modified (PTM)	CEP	C-terminally encoded peptide	Signal	21
	CLE	Clavata/Embryo Surrounding Region	Signal	43
	GLV/RGF/CLEL	Golven/Root Growth Factor	Signal	12
	IDA	Inflorescence Deficient in Abscission	Signal	8
	PIP	PAMP-induced Secreted Peptide	Signal	4
	PSK	Phytosulfokine	Signal	8
	PSY	Plant Peptide Containing Sulfated Tyrosine	Signal	11
Cysteine rich	2SA	2S Albumin	Antimicrobial	2
	ECL	Egg Cell 1-Like	Signal	10
	EPFL	Epidermal Patterning Factor-Like	Signal	12
	GASA	Gibberellic Acid Stimulated in *Arabidopsis*	Signal	20
	HEVEIN	Hevein	Antimicrobial	9
	Kunitz	Kunitz-P trypsin inhibitor	Peptidase inhibitor	17
	LAT52-POE	LAT52/Pollen Ole e 1 Allergen	Signal	19
	MEG	Maternally Expressed Gene	Signal	2
	N26	Nodulin26	Signal	1
	nsLTP	non-specific Lipid Transfer Protein	Signal	122
	PCY	Plantcyanin/Chemocyanin	Signal	46
	PDF	Plant Defensin	Antimicrobial	51
	RALF	Rapid Alkalinization Factor	Signal	11
	RC	Root Cap	Signal	2
	STIG-GRI	Stigma1/GRI	Signal	10
	T2SPI	Potato type II proteinase inhibitor	Peptidase inhibitor	13
	THL	Thionin-like	Antimicrobial	18
	TPD	Tapetum Determinant 1	Signal	6
	Kaz	Kazal family inhibitors	Peptidase inhibitor	2
	PDL	Plant Defensin-like	Antimicrobial	2
	LCR	Low-molecular weight Cys-rich	Unknown	3
	TAX	Taximin	Signal	3
	SCR/SP11	S-locus Cysteine Rich	Signal	4
Non-Cys-rich/Non-PTM	CTLA	Cytotoxic T-lymphocyte antigen-2 alpha	Peptidase inhibitor	5
	GRP	Glycine-rich Protein	Unknown	6
	PhyCys	Phytocystatin	Peptidase inhibitor	9
	PNP	Plant Natriuretic Peptide	Signal	7
	PRP669	Pro-rich Protein Group 669	Unknown	4
	Subln	Subtilisin inhibitor	Peptidase inhibitor	17
Functional Precursor	CAPE	CAP-derived Peptide	Signal	14
	MtSUBPEP	Subtilisin-embedded Plant Elicitor Peptide	Signal	3
	Total			557

**Fig. 2:**
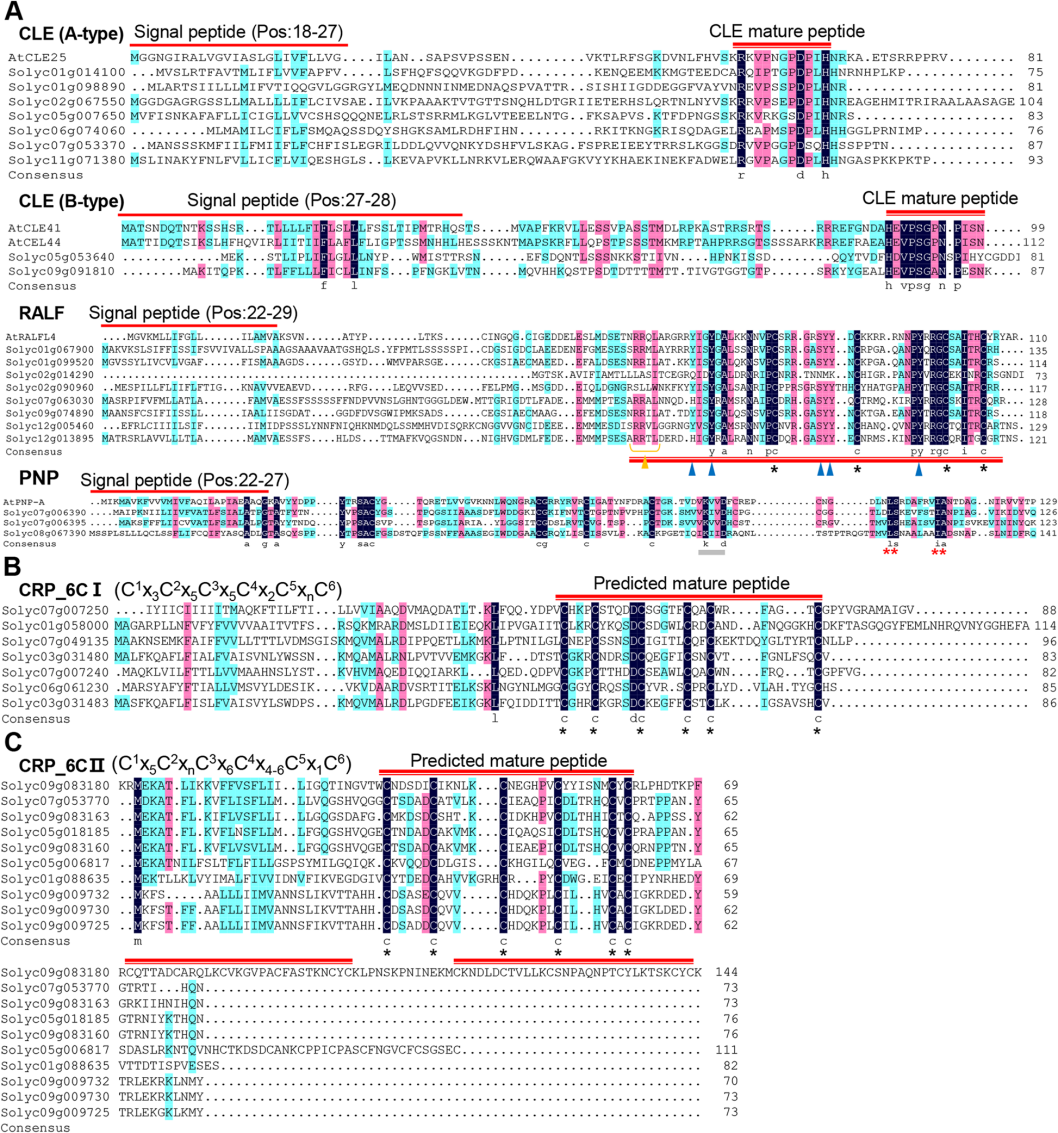
Structural characteristics of proproteins of typical SlSSPs. **A** The multiple sequence alignment of typical SSP families. CLE represents PTM peptides, RALF represents CRPs, PNP represents Non-Cys-rich/No-PTM peptides. **B**, **C** Two putative new SSP families predicted by manual analysis. Red single lines indicate signal peptides, and red double lines indicate putative mature peptides. Yellow arrows indicate the putative endoprotease dibasic cleavage sites (RR), and blue arrows indicate the conserved tyrosine residues. Gray underlines indicate the “K(V/I)(V/I)D” domain and red stars indicate the conserved “LS” and “IA” domains in (**A**). Black stars indicate the conserved cysteine residues. Brackets represent mature peptide sequence modules of SSP families from (**B**, **C**). Superscript and subscript numbers represent position of cysteine residues and the number of amino acids, respectively.“n” indicates any number

The identified SlSSPs covered most of the known peptide families. Totally we identified 107 PTM family members, including CEP, CLE, Golven/Root Growth Factor (GLV/RGF/CLEL), IDA, PIP, PSK and PSY; 385 CRP family members, including ECL, EPFL, GASA, HEVEIN, Kunitz, LAT52-POE, nsLTP, PCY, PDF, RALF, T2SPI, THL, etc.; 48 Non-Cys-rich/Non-PTM family members, including CTLA, GRP, PhyCys, PNP, PRP669 and Subln (Table 1). The CLE peptide family is one of the largest peptide families in tomato, with a total of 43 members, which are further classified into A-type and B-type according to the classification criteria of *Arabidopsis* (Whitford et al. 2008). Similar to *Arabidopsis* CLEs, most of the SlCLEs belong to A-type, and only seven SlCLEs are B-type. All the SlCLEs share similar conserved residues as *Arabidopsis* CLEs (Fig. 2A). Most of the tomato cysteine-rich peptides (CRPs) contain 2–12 cysteine residues at the C-terminal region of the preproproteins, where they form intramolecular disulfide bonds to resist proteolytic digestion. One typical CRP family, the RALF family, was identified in tomato to contain 11 members with four conserved cysteine residues and putative endoprotease dibasic cleavage sites (RR), as identified by Pearce et al. (Pearce et al. 2001) (Fig. 2A). PNP is a Non-Cys-rich/Non-PTM family. Seven PNP members were identified in tomato, which share the similar conserved residues as AtPNP-A with distinct sequence domains “K(V/I)(V/I)D” and “LSXXA(F/I)XXIA” (Ludidi et al. 2002) (Fig. 2A).

Considering that half of the SlSSPs are unknown, we tried to identify new SSP families. Two potentially novel CRP families with a conserved domain of six cysteine residues were identified by analysis of the C-terminal sequences of the non-classified SlSSPs, named CRP_6C I and CRP_6C II, containing seven and ten members respectively (Fig. 2B, C). The potential mature peptides of CRP_6C I and CRP_6C II families are distinct from all known CRPs, and the BLAST results showed that no similar peptides were found in other plant species. Interestingly, all CRP6_C I members contain only one conserved peptide domain (C^1^x_3_C^2^x_5_C^3^x_5_ C^4^x_2_ C^5^x_n_C^6^), while some CRP6_C II members contain 2–3 conserved domains (C^1^x_5_C^2^x_n_C^3^x_6_C^4^x_4-6_C^5^x_1_C^6^). Further study is needed to verify the new peptide families and to analyze their functions.

### Identification of unannotated small secreted peptides in tomato

Genes encoding small secreted peptides can be easily missed during genome annotation due to their small size, and small open reading frames (sORFs) are often ignored during SSP identification. Our TBLASTN results showed that many SlSSP-encoding genes are unannotated. In order to ensure the comprehensiveness of SlSSP identification, the sORFs (25–250 aa) from non-coding sequences (NCDS) on the 12 tomato chromosomes were identified using the ORF finder (https://www.ncbi.nlm.nih.gov/orffinder/). The results showed that 3,175,518 sORFs were found with 25–250 aa in length, among which 61,306 sORFs have the characteristics of putative SSPs based on the presence or absence of N-terminal signal peptides, transmembrane domains and whether the sORF encodes an ER docking protein (Table 2, Supplementary Table S2). Most (76.4%) of the putative SSP-encoding sORF are 25–50 aa in length (Supplementary Fig. S1A). Considering the decline in the amount of annotated SlSSPs less than 50 aa in tomato (Supplementary Fig. S1B, Fig. 1B), sORFs have been largely overlooked in tomato genome annotation and these sORFs could be a great complement to genes encoding SlSSPs.

**Table 2:** Bioinformatic identification and filtering of tomato peptide-encoding sORFs

Tomato Chromosome	No. sORFs kept after each sequential filter				
	**25–250 aa**	**N-terminal SP**	**Non-TM**	**Putative SSP**	**Known SSP families**
0	48,555	590	570	570	0
1	362,481	7,262	7,064	7,064	9
2	196,102	3,884	3,796	3,794	6
3	251,100	4,800	4,668	4,668	6
4	258,114	5,108	4,998	4,998	2
5	274,533	5,583	5,433	5,432	3
6	179,709	3,503	3,410	3,409	2
7	279,966	5,515	5,383	5,382	8
8	263,811	5,203	5,076	5,076	6
9	287,462	5,986	5,840	5,840	3
10	273,707	5,573	5,447	5,447	2
11	217,273	4,320	4,200	4,200	6
12	282,705	5,555	5,426	5,426	6
Total	3,175,518	62,882	61,311	61,306	59

*sORFs* small ORFs, *SP* Signal peptide, *TM* Transmembrane

Next, the 61,306 putative SSP-encoding sORFs were predicted in MtSSPdb. Unexpectedly, only 59 sORFs (0.1%) were grouped into 18 known SSP families. This may suggest that there are still many unknown peptide families to be discovered. On the other hand, a large number of the sORF predictions might be false positive due to lack of annotation. For example, ORF57_*Solyc03g006020*-*Solyc03g006030* was identified as a SSP-encoding sORF through the above multi-step ORF screening and classified as a member of the CEP family. However, gene prediction result by FGENESH (http://www.softberry.com/berry.phtml?topic=fgenesh&group=programs&subgroup=gfind) showed that no potential gene deriving from this ORF was predicted, indicating that it is a false-positive result. Further analysis based on genome re-annotation, RNA-seq and mass spectrometry is needed to confirm these sORFs.

### Expression patterns of *SlSSPs* in different tissues and under drought stress

**Fig. 3:**
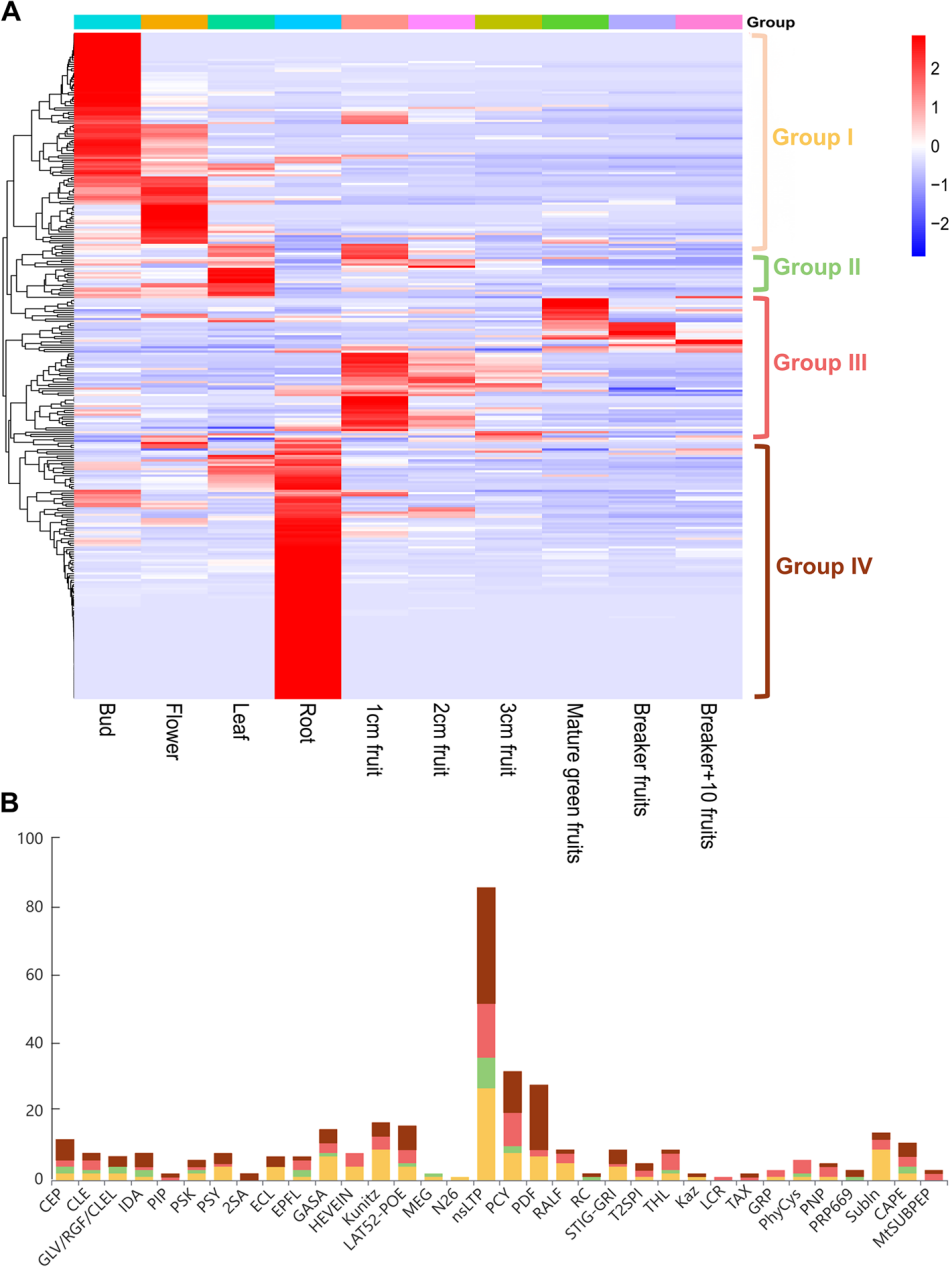
The expression pattern of tomato *SSP* genes in different tissues. **A** Heatmap visualization of the expression pattern of tomato *SSP* genes in bud, flower, leaf, root, 1 cm fruit, 2 cm fruit, 3 cm fruit, mature green fruits, breaker fruits and breaker + 10 fruits. The expression data was downloaded from Tomato Expression Database (D004, http://ted.bti.cornell.edu/). The heatmap was generated by TBtools. **B** The number of SSP members of different families in group I (yellow), group II (green), group III (red), and group IV (brown)

To obtain the expression information of the *SlSSP* genes in different tissues, we searched the public tomato transcriptome database (D004, http://ted.bti.cornell.edu/). The expression level of 449 *SlSSPs* from known SSP families in 10 different tissues was obtained and visualized (Fig. 3A). Most of the *SlSSPs* are highly expressed in roots, some are expressed in flowers and fruits, only a small number of *SlSSPs* are specifically expressed in leaves (Fig. 3A). The *SlSSPs* were divided into four groups based on their expression patterns. Members of some *SSP* families are expressed in all tissues, including *CEP*, *CLE*, *IDA*, *PSK*, *EPFL*, *GASA*, *LAT52-POE*, *nsLTP*, *PCY*, *THL* and *CAPE*. Genes encoding members of the PSY, Kunitz, PDF, RALF, STIG-GRI, T2SPI, PNP, Subln families are expressed in most tissues except leaves (Fig. 3B), suggesting functional diversity of the *SlSSPs*.

**Fig. 4:**
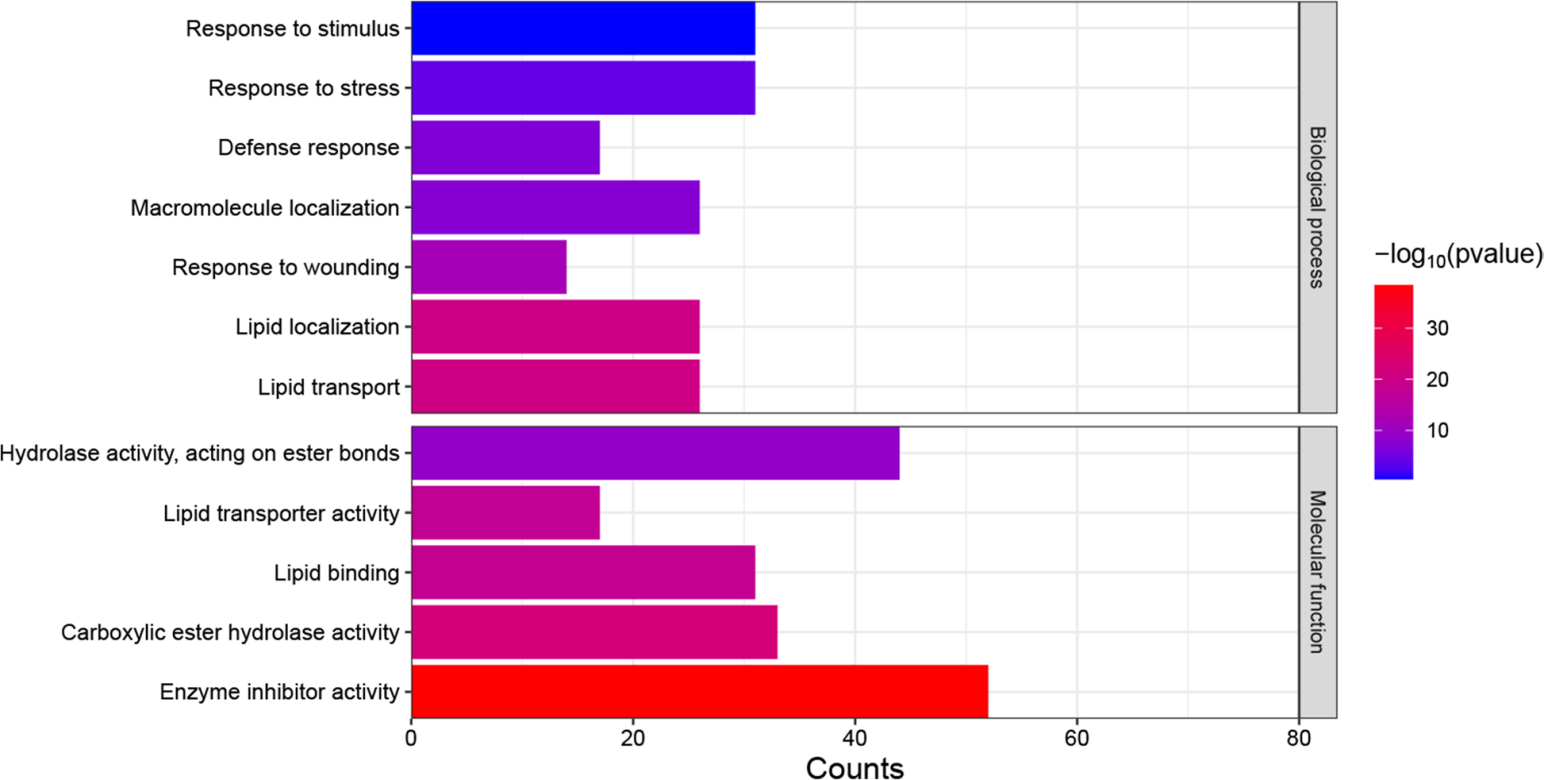
GO analysis of tomato *SSPs*. Go terms involved in stress response. X-axis represents the number of genes in each GO Term, Y-axis is GO Terms. The color indicates P value. Go analysis was done by AgriGO v2.0 (http://systemsbiology.cau.edu.cn/agriGOv2/)

To characterize the molecular biological functions of the SlSSPs, we performed GO analysis for the SlSSP-encoding genes using the singular enrichment analysis (SEA) tool from the agriGO online tool (http://systemsbiology.cau.edu.cn/agriGOv2/). The results showed that 445 out of the 1,050 *SlSSPs* had GO annotations, and 23 GO terms were significant (*P* value ≤ 0.001 and FDR ≤ 0.05), including lipid transport, lipid localization, peptidase inhibitor activity, peptidase regulator activity and nutrient reservoir activity. Additionally, a number of the SlSSP-encoding genes were enriched in stress response GO terms such as response to wounding, response to stress, defense response, and response to stimulus (Fig. 4, Supplementary Table S3), suggesting potential roles of SlSSPs in response to abiotic stresses.

**Fig. 5:**
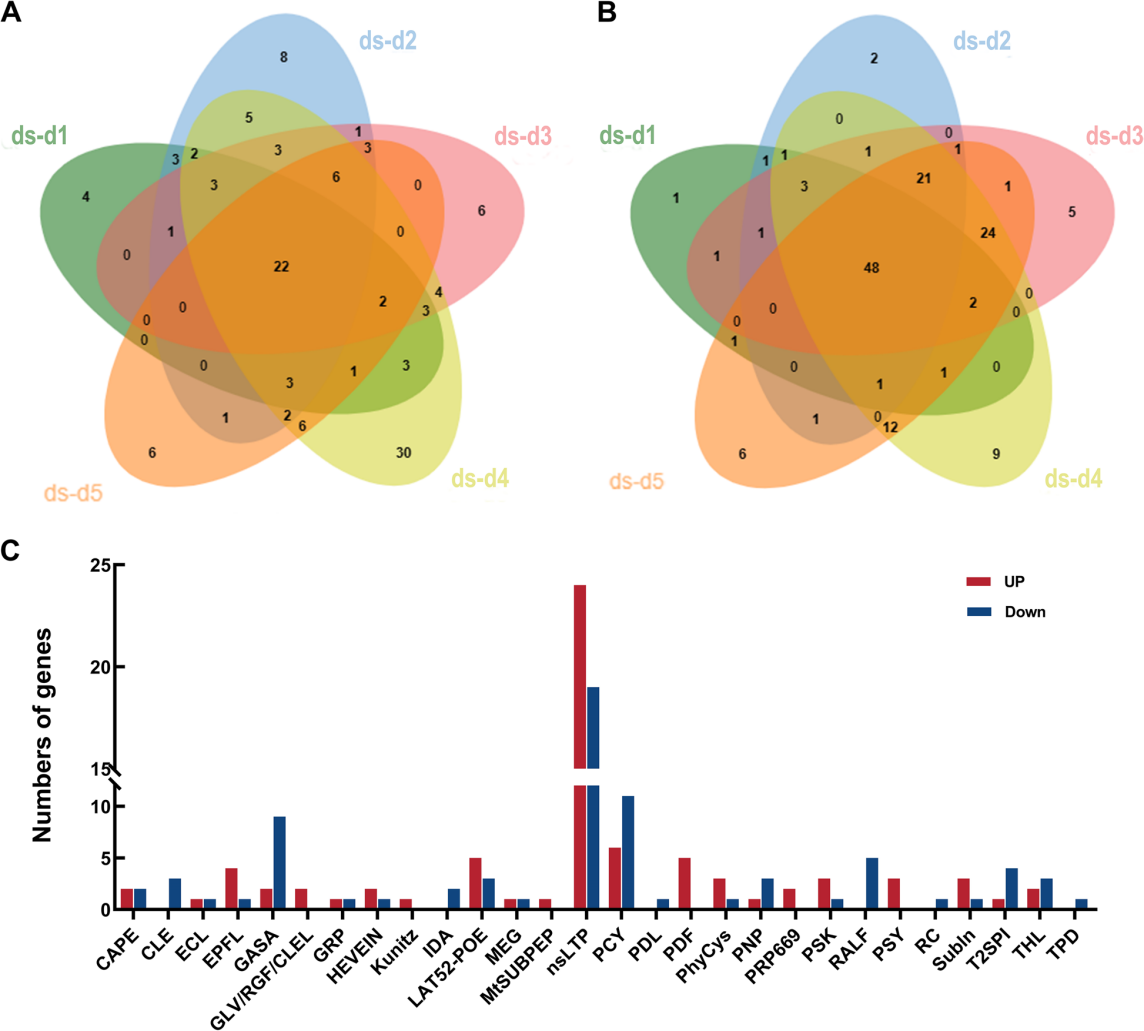
Expression analysis of *SlSSPs* under drought stress. **A** Up-regulated *SlSSPs* in drought stress 1 day (ds-d1), 2 days (ds-d2), 3 days (ds-d3), 4 days (ds-d4) and 5 days (ds-d5) after treatments. Fold change = FPKM_ds-dn_/FPKM_ck_, Log2Foldchange ≥ 1 and qvalue ≤ 0.05. **B** Down-regulated *SlSSPs* in drought stress 1 day (ds-d1), 2 days (ds-d2), 3 days (ds-d3), 4 days (ds-d4) and 5 days (ds-d5) after treatments. Fold change = FPKM_ds-dn_/FPKM_ck_, Log2Foldchange ≤ -1 and qvalue ≤ 0.05. **C** Up- and down-regulated members of *SlSSP* families under drought stress

We further analyzed the expression pattern of the *SlSSP* genes under drought stress using the RNA-seq data from NCBI (GSE151277). 128 SlSSP-encoding genes were found to be significantly up-regulated at different stages of drought stress treatments (ds) (Log2Foldchange ≥ 1 and qvalue ≤ 0.05), among which 47, 63, 54, 95 and 52 *SlSSP*s are up-regulated 1 day, 2 days, 3 days, 4 days and 5 days after treatments, respectively (Fig. 5A). Similarly, 144 SlSSP-encoding genes were significantly down-regulated at different stages of drought stress, among which 61, 81, 108, 123 and 119 *SlSSP*s are down-regulated 1 day, 2 days, 3 days, 4 days and 5 days after treatments, respectively (Fig. 5B). Totally 75 *SlSSPs* from 28 SlSSP families were specially up- or down-regulated under drought stress, including members of CAPE, CLE, ECL, EPFL, GASA, GLV, GRP, HEVEIN, Kuntiz, IDA, LAT52-POE, MEG, MtSUBPEP, nsLTP, PCY, PDF, PDL, PNP, PhyCys, PRP669, PSK, PSY, RALF, RC, Subln, T2SPI, TPD and THL families (Fig. 5C). These results suggested that a large number of the tomato SSPs might be involved in drought stress response.

### Potential roles of tomato CEP family members in drought response

**Fig. 6:**
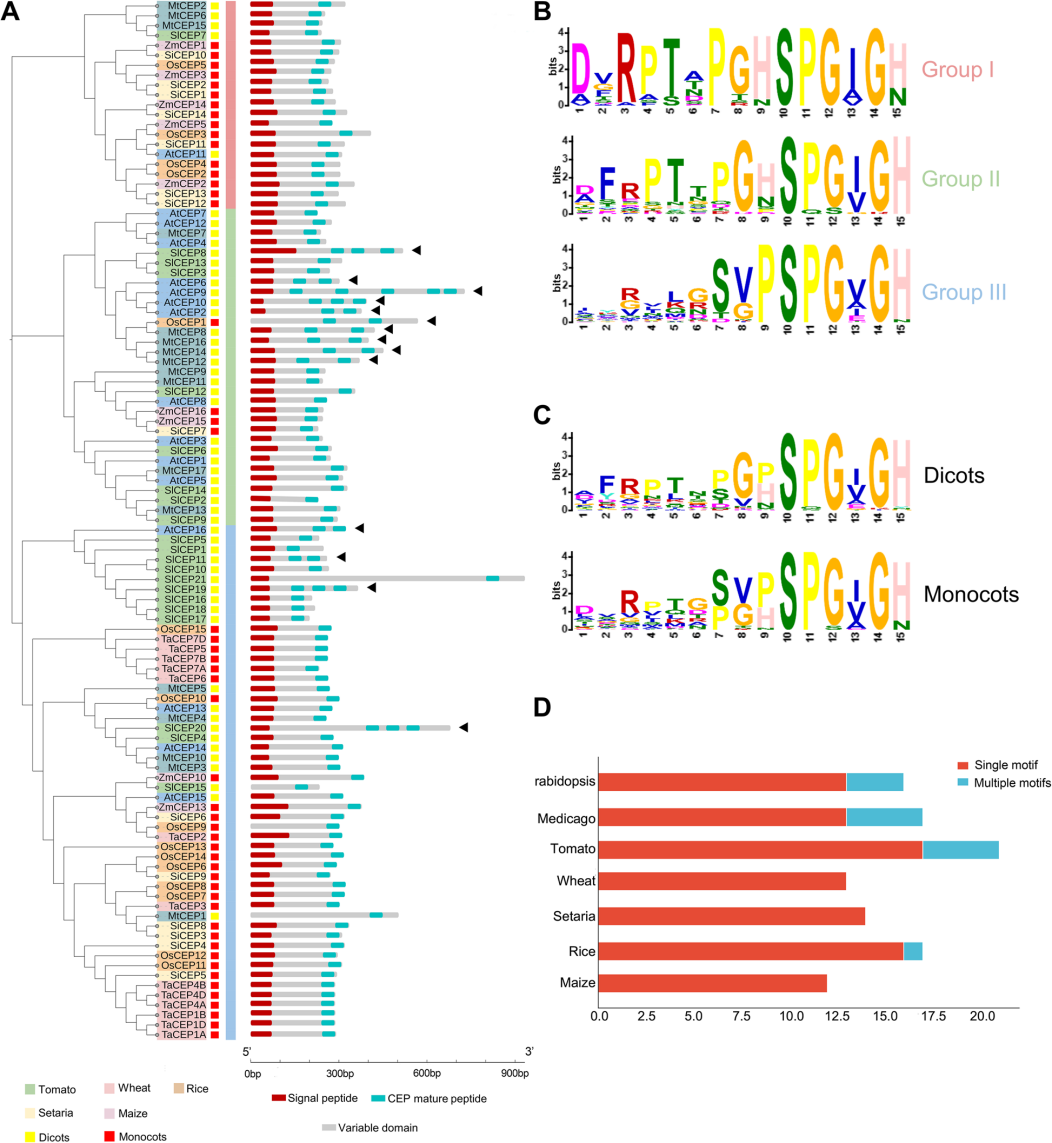
Identification of CEP family members in tomato. **A** Phylogenetic tree and protein structure of AtCEPs, SlCEPs, MtCEPs, TaCEPs, OsCEPs, SiCEPs and ZmCEPs. The alignment was performed using Muscle with the full length protein sequences. The phylogenetic tree was constructed by the Maximum Likelihood method with 1000 bootstrap replications. The phylogenetic tree was drawn by the iTOL online tool (https://itol.embl.de/itol.cgi). The protein structures of CEPs were constructed by IBS software. Red boxes indicate monocotyledon plants, and yellow boxes indicate dicotyledon plants. Different colors indicate different species. Red boxes represent signal peptide, blue-green boxes represent CEP mature peptide, and gray boxes represent variable domain, “◀” represents CEP proteins containing multiple CEP domains in protein structure. **B**, **C** Logos were created by the MEME online tool (https://meme-suite.org/meme/tools/meme). **D** The number of single-domain genes (red) and multiple-domains genes (blue) in *Arabidopsis*, *Medicago*, wheat, *Setaria*, rice, maize and tomato

Previous studies have showed that a large number of *CEP* genes can be induced by abiotic stresses, and some CEPs are involved in ABA signaling and plant stress responses in *Arabidopsis*, *Setaria* and *Triticum* (Smith et al. 2020; Zhang et al. 2021; Tian et al. 2022; Taleski et al. 2023). In this study, a total of 21 *SlCEP* genes (4 unannotated) were identified. A phylogenetic tree of CEP precursor proteins from *Arabidopsis*, *Medicago*, wheat, *Setaria*, rice, maize and tomato was constructed and the CEP members were clustered together in three branches, with each branch including peptides from both monocotyledon and dicotyledon plants (Fig. 6A). Protein structure analysis of the CEPs showed that *SlCEP8*, *SlCEP11*, *SlCEP19* and *SlCEP20* encode more than one CEP peptides (Fig. 6A). It’s interesting to note that *CEP* genes in monocotyledon plants tend to encode single mature CEP peptide, while more *CEP* genes in dicotyledon plants encode multiple mature CEP peptides (Fig. 6A, D). A conserved ‘SPGXGH/N’ domain was identified within the CEP domain of all the CEP proteins analyzed in this study (Fig. 6B, C). The results from multiple sequence alignments using the CEP domain sequences supported the above mentioned classification based on full length precursors (Supplementary Fig. S2B, C). Chromosomal distribution analysis showed that 21 of the *SlCEPs* were mapped on 4 chromosomes, including Chr1, Chr2, Chr3 and Chr7 (Supplementary Fig. S2A). Four gene clusters were found on Chr2, 3 and 7, including *SlCEP2-3* and *SlCEP6-9* on Chr2, *SlCEP12-14* on Chr3, and *SlCEP15-21* on Chr7. These gene clusters of *SlCEPs* are likely the result of tandem replication events.

To study potential roles of the *SlCEPs* in plant stress response, we analyzed the *cis*-acting elements in the promoter regions between -2 kb to + 1 bp upstream of the transcription start sites. The results revealed the presence of large number of *cis*-elements related to abiotic stress response on *SlCEP* promoters, including the antioxidant-responsive element ARE, the defense and stress-responsive element TC-rich repeats, the dehydration-responsive element DRE/MBS, and the low temperature-responsive element LTR. Multiple hormone-responsive elements were also found in the promoter regions of the *SlCEP*s, including the abscisic acid-responsive element (ABRE), the MeJA-responsive element (TGACG and CGTCA-motif) and the ethylene-responsive element (ERE). Among them, the cis-acting element DRE can work together with the transcription factor DREB which is tightly associated with drought tolerance (Sakuma et al. 2006). The ABRE element can be involved in perceiving ABA-mediated osmotic stress signals and regulating drought-responsive genes (Kim et al. 2011).

**Fig. 7:**
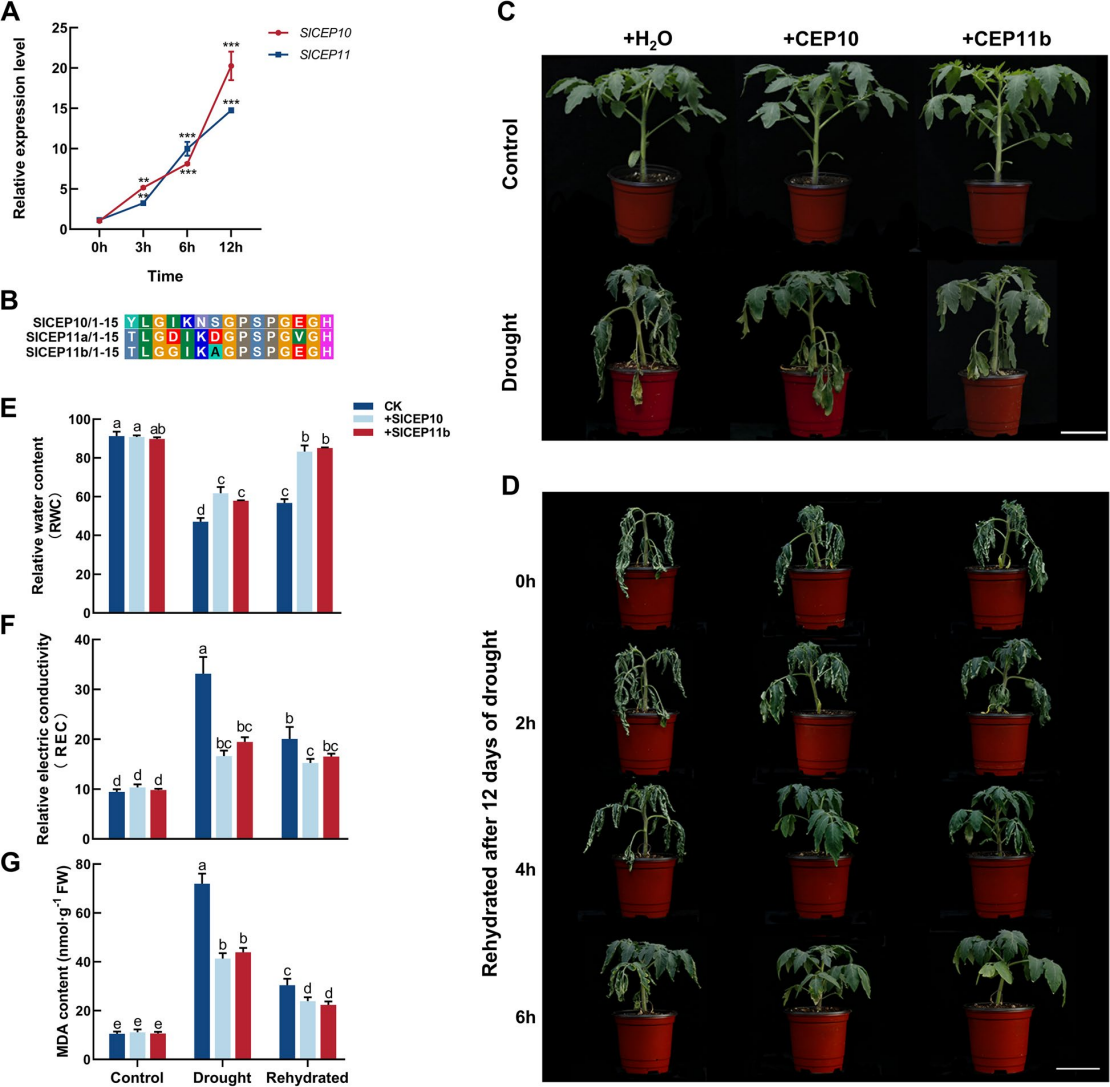
Exogenous application of SlCEP10 and SlCEP11b peptides altered drought response in tomato. **A** Expression level of *SlCEP10*, *SlCEP11* under drought (20% PEG6000) stress treatments for 0, 3, 6 and 12 h based on qRT-PCR. qRT-PCR was performed with three biological replicates and three technological replicates. The data represent mean ± SD, * *p* < 0.05, ** *p* < 0.01, *** *p* < 0.001 was determined by ordinary one-way ANOVA. * indicates a significant difference at the *P* < 0.05 level. **B** The sequences of synthesized SlCEP10, SlCEP11a and SlCEP11b peptides and their sequence alignment results. **C** 4-week-old tomato plants treated with 5 µM SlCEP10 or SlCEP11b peptide for 8 days under control and drought conditions. **D** 4-week-old tomato plants treated with 5 µM SlCEP10 or SlCEP11b peptide for 12 days under drought conditions and 1, 2, 4, 6 h after rehydration. **E**–**G** Measurement of relative water contents (RWC) (**E**), relative electric conductivity (REC) (**F**) and malondialdehyde (MDA) contents (**G**), respectively 8 days after drought condition. Sample size *n* = 15 in (**E**–**G**). All statistics analyses were performed with three biological replicates. Different lowercase letters in (**E**–**G**) indicate statistically significant differences based on ordinary one-way ANOVA (*p* < 0.05). Bar = 6 cm in (**C**, **D**)

The transcript levels of the *SlCEP* genes were further determined by qRT-PCR in 15-day-old tomato leaves treated with 20% (m/v) PEG 6000 for drought stress mimicking. Most of the *SlCEP* genes were differentially expressed at some stages of the PEG treatments. The expression of *SlCEP12* and *SlCEP16* were significantly repressed by PEG treatments. The expression of *SlCEP8* and *SlCEP14* were slightly increased at 3 h and 6 h, and decreased at 12 h after treatments. *SlCEP1*, *SlCEP2*, *SlCEP3*, *SlCEP4*, *SlCEP5*, *SlCEP7*, *SlCEP13*, *SlCEP15*, *SlCEP18*, *SlCEP19*, *SlCEP20* and *SlCEP21* were slightly up-regulated at some time after PEG treatments. *SlCEP6*, *SlCEP9* and *SlCEP17* were less responsive to PEG treatments. It is worth noting that the expression of *SlCEP10* and *SlCEP11* was significantly induced by PEG treatments (Fig. 7A, Supplementary Fig. S3).

### Exogenous application of SlCEP10 and SlCEP11b peptides enhanced drought tolerance of tomato plants

To study the role of SlCEP10 and SlCEP11 peptides in drought stress response, predicted CEP peptides from *SlCEP10* (*Solyc02g092890*) and *SlCEP11* (*Solyc03g044180*) were synthesized for in vitro treatments. The SlCEP11 precursor protein contains two conserved CEP domains with high homology and SlCEP11b was synthesized for peptide treatments in this study (Fig. 7B). Drought stress treatments were performed via water withdrawal and synthetic peptides were sprayed on leaf surface to test their effects on drought tolerance. In comparison with the stressed tomato plants that were sprayed with water, after eight days of drought treatments, the plants sprayed with SlCEP10 or SlCEP11b peptides showed significantly better performance (Fig. 7C), with more stretched leaves, firmer stalks and higher relative water content (RWC) (Fig. 7E). Exogenous application of SlCEP10 or SlCEP11b peptides did not cause detectable difference on well-watered tomato plants. With the increase of concentration, the alleviating effect of SlCEP10 peptides on drought stress increased first and then decreased, which was consistent with the common dose–effect of small peptides (Figure S4). 5 µM SlCEP10 peptides significantly alleviated the damage to tomato plants caused by drought treatments and was used in the following experiments.

The results of relative electric conductivity (REC) and MDA measurements of tomato leaves showed that both SlCEP10 and SlCEP11b peptide treatments significantly decreased the electrolyte leakage and MDA accumulation in tomato leaves under drought stress (Fig. 7F, G). 12 days after drought treatments, we preformed rehydration on these tomato plants and found that plants treated with SlCEP10 or SlCEP11b peptides recovered faster, almost getting back to normal conditions 4 h after rehydration (Fig. 7D). We also measured the RWC, REC and MDA contents in leaves 6 h after rehydration and the results indicated that tomato plants recovered better with SlCEP10 or SlCEP11b peptide treatments (Fig. 7E-G). These results suggested that exogenous application of SlCEP10 or SlCEP11b peptides enhanced the drought tolerance of tomato plants.

**Fig. 8:**
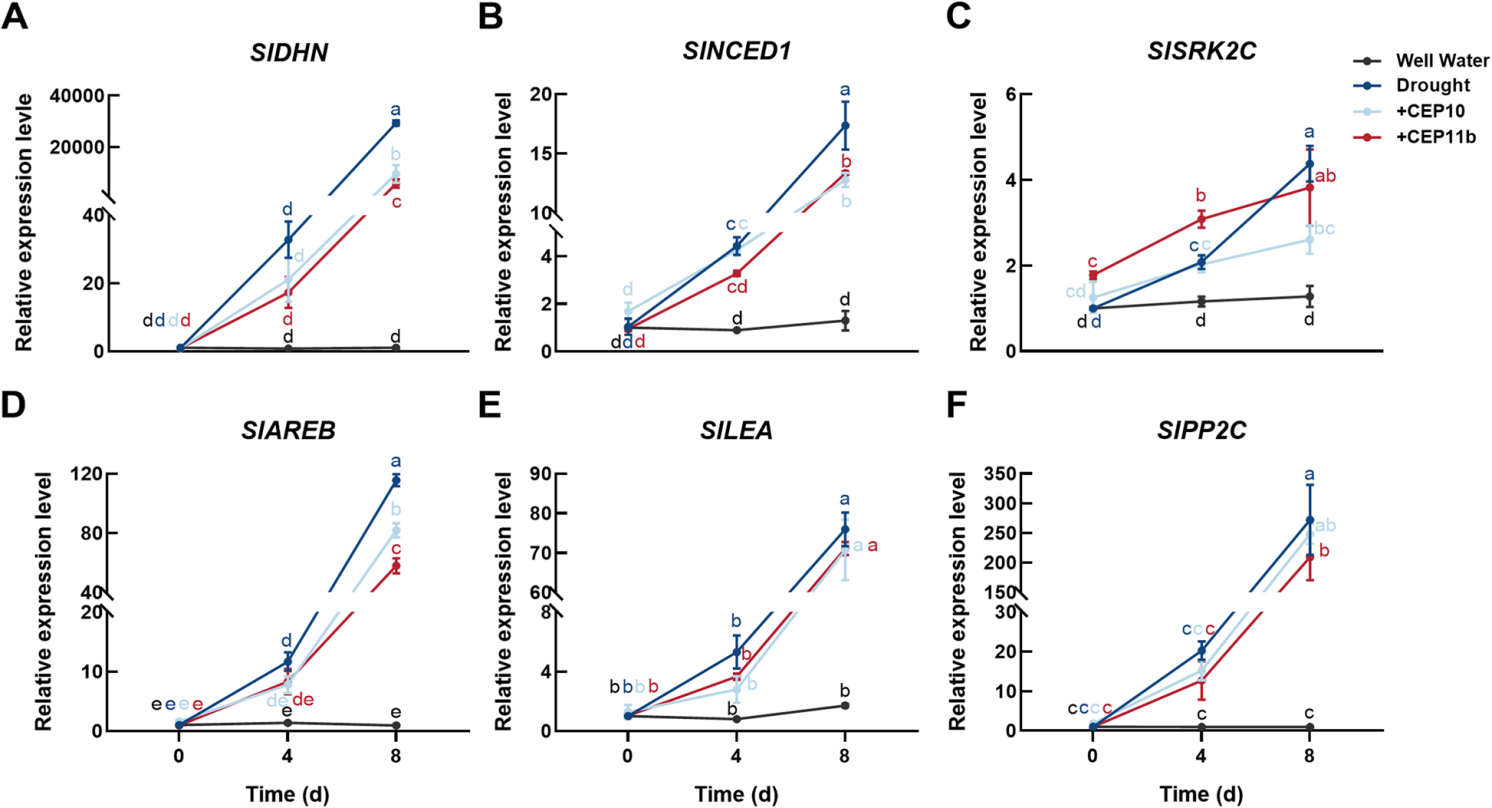
Relative expression levels of drought-related genes under drought stress after exogenous application of SlCEP10 or SlCEP11b peptides. Relative expression levels of *SlDHN* (**A**), *SlNCED1* (**B**), *SlSRK2C* (**C**), *SlAREB* (**D**), *SlLEA* (**E**) and *SlPP2C* (**F**) by qRT-PCR. qRT-PCR was performed with three biological replicates and three technological replicates. Different lowercase letters indicate statistically significant differences based on ordinary one-way ANOVA (*p* < 0.05)

Earlier studies showed that tomato genes *SlDHN* (*Solyc02g084850*), *SlNCED1* (*Solyc07g056570*), *SlSRK2C* (*Solyc04g012160*), *SlAREB* (*Solyc04g078840*), *SlPP2C* (*Solyc03g096670*) and *SlLEA* (*Solyc01g095140*) could be induced by drought stress (Sun et al. 2011; Bolger et al. 2014; Landi et al. 2017; Wang et al. 2018; Jia et al. 2022; Qiao et al. 2022). We then determined the expression changes of these drought-responsive genes after peptide treatments. In consistent with the phenotypic changes, expression levels of these genes increased significantly after drought stress treatments, but the increase in expression levels was inhibited by SlCEP10 or SlCEP11b peptide treatments (Fig. 8A-F). This result further supported the conclusion that that SlCEP10 and SlCEP11b could alleviate the damage caused by drought stress in tomato.

We also determined the transcript levels of *SlCEP10* and *SlCEP11* in salt stress (200 mM NaCl) and ABA treatments (100 µM ABA) by qRT-PCR. The results showed that *SlCEP10* and *SlCEP11* were slightly induced by ABA and significantly induced by salinity (Supplementary Fig. S5B, C), suggesting potential roles of these peptides in response to other abiotic stresses.

## Discussion

### Identification of annotated and un-annotated SSPs

In this study, we identified 1,050 putative SlSSPs, which constitutes around 3% of all annotated genes in tomato. 557 SlSSPs were classified into 38 known SSP families by MtSSPdb prediction (80%), CD-search (1%), BLAST search (7%) and HMM search (12%) (Supplementary Table S1). Not all the SSP family members can be predicted by BLAST search of public databases. HMM search as well as manual verification are essential approaches to conduct thorough and in-depth analysis of a specific SSP family (Zhang et al. 2021). Half of the putative SlSSPs belong to unknown families (Fig. 1D), which needed further identification and classification. 149 (30%) of the unclassified SlSSPs are CRPs, among which two novel SlCRP families were identified in this study by comparing the number and position of cysteine residues in their protein sequences, named CRP_6C I and CRP_6C II (Fig. 2B, C). Furthermore, through TBLASTN analysis, we identified a number of unannotated SSPs in CEP, CLE and PIP families, suggesting that the current genome annotation of the tomato genome was inadequate for complete identification of SlSSPs. Some known SSPs, including systemin (encoded by Solyc05g051750), were not included in the SlSSPs identified in this study due to the lack of a signal peptide in the systemin precursor protein (Pearce et al. 1991), which means bioinformatic approaches for SSPs identification used in this study can only obtained SSPs with common structural features. In-depth analysis including peptidome profiling were needed for studying a specific SSP family.

Similar to *Arabidopsis* (Lease and Walker 2006), we also found a skew in the protein length frequency distribution in the tomato genome (Supplementary Fig. S1B). The results of this study indicated an incomplete annotation of genes in the tomato genome because the small-size proteins are easily missed in genome annotation. We thus analyzed small open reading frames on the tomato genome and identified 61,306 putative sORF-encoded SSPs (Table 2). Since some peptides are encoded by genes with multiple exons (Carbonnel et al. 2022), the results of the sORF analysis based on single-exon ORFs are not complete. This can be improved by whole genome re-annotation. After classifying the sORFs via MtSSPdb, we noticed that some of the sORFs were not real protein-coding genes, indicating that the presence of false positive results of sORFs. Multi-omics joint analysis can be done to help in accurate identification of sORF-encoding SSPs.

### Potential function of SlSSPs in stress response

The result of GO analysis showed that some *SlSSPs* from HEVEIN, PDF and Subln families might be involved in stress response (Fig. 4). Only 445 out of the 1,050 putative *SlSSPs* had GO annotations due to the limited tomato GO database with 20,036 annotated genes in 3,947 GO terms (Tian et al. 2017). We also analyzed the expression pattern of *SlSSPs* under drought stress and found that 128/144 *SlSSP*s were significantly up/down-regulated (Fig. 5B, C). It’s interesting to note that only a few *SlSSP* genes were highly expressed in leaves (Fig. 3A). SSP families such as CLE, CEP, RALF and PIP, which have been reported to be involved in abiotic stress (Atkinson et al. 2013; Takahashi et al. 2018; Tian et al. 2022; Zhou et al. 2022), showed low expression levels. In the online resource for tomato transcriptome analysis (GSE151277, https://www.ncbi.nlm.nih.gov/geo/), the expression levels of tomato genes were examined 1, 2, 3, 4 and 5 days after drought treatments. Some *SlSSP*s that are induced at earlier stages after treatments could be missed in this database.

Compared with model plants like *Arabidopsis*, rice and wheat, the limited function annotation and public expression database restrict our exploration of SlSSPs in stress response. qRT-PCR analysis of the *SlSSP* genes under drought, salt, heat and cold stresses will provide more information. In addition, exogenous application of synthesized peptides is also an efficient way in identifying stress-related peptides.

### Tomato CEP peptide family members may be involved in drought stress response

CEP peptides have been reported to be involved in root and shoot growth and development, as well as regulation of nitrogen acquisition in *Arabidopsis* (Roberts et al. 2013; Tabata et al. 2014; Ohkubo et al. 2017; Taleski et al. 2018). Recent studies have showed that CEP peptides are also involved in abiotic stress response and ABA signaling (Smith et al. 2020; Zhang et al. 2021; Tian et al. 2022). In this study, we found that exogenous application of SlCEP10 or SlCEP11b peptides significantly improved drought stress tolerance of tomato plants without affecting plant growth under normal conditions (Fig. 7C-G). Biosynthesized peptides have been reported to be applied in modern agriculture with better specificity compared to phytohormones which are often involved in multiple development and stress response processes (Zhang and Gleason 2020). Biosynthesis of SlCEP10 and SlCEP11b peptides with significantly lower cost would facilitate the application of these peptides in agriculture.

Further characterization of the function of these two peptides in drought response will provide more mechanistic information about how they act in altering the stress response process of tomato plants. In addition to the drought-tolerant phenotypes, 6 drought-induced genes were found up-regulated in peptide-treated plants (Fig. 8). Further study of the downstream gene network of the CEP signaling will provide detailed information about CEP-mediated tomato drought stress response. It will be also interesting to identify the corresponding receptor of the SlCEP peptides which initiates signal transduction. CEPR1, CEPR2 from the LRR-RLK family have been reported to function as CEP receptors in *Arabidopsis* in regulating lateral root initiation (Tabata et al. 2014). Zhang et al. revealed that *AtCEPR1* and *AtCEPR2* also function in mediating ABA response to balance plant growth and abiotic stress responses (Zhang et al. 2021). We have identified three potential tomato CEP receptors including SlCEPR1, SlCEPR2 and SlCEPR2-like (encoded by *Solyc04g077010*, *Solyc11g020280* and *Solyc06g065260*) on the tomato genome. Whether these receptors are involved in CEP-mediated drought response in tomato needs to be explored.

## Materials and methods

### Identification and classification of small secreted peptides in tomato

All protein sequences of tomato (*Solanum lycopersicum*) SSPs were downloaded from Phytozome (https://phytozome-next.jgi.doe.gov/) (Goodstein et al. 2012) and the Sol Genomics Network (https://solgenomics.net/) (Fernandez-Pozo et al. 2015). Based on the common structure and sequence features of known plant peptides, a multi-step procedure was used to identify tomato SSPs as described in Fig. 1A: Firstly, all the proteins in 25–250 amino acids were obtained; Secondly, the SignalP-5.0 software (https://services.healthtech.dtu.dk/service.php?SignalP) (Almagro Armenteros et al. 2019) was used to predict N-terminal signal peptides, and proteins without an N-terminal signal peptide were removed from the list; Thirdly, transmembrane (TM) domains were predicted using the TMHMM v2.0 software (https://services.healthtech.dtu.dk/ service.php?TMHMM-2.0) (Krogh et al. 2001) to remove membrane proteins; Fourthly, putative endoplasmic reticulum docking proteins were eliminated by identifying the C-terminal conserved domain K/HDEL (Lys/His-Asp-Glu-Leu) (Napier et al. 1992). For SlSSPs including multiple transcripts, the longest transcript was used for further analysis.

The putative SlSSPs were predicted and classified into different known SSP families using the *Medicago truncatula* Small Secreted Peptide Database (MtSSPdb, http://mtsspdb.noble.org/database/) (Boschiero et al. 2020) and CD-search (https://www.ncbi.nlm.nih.gov/Structure/cdd/wrpsb.cgi) (Lu et al. 2020) based on their homology with HMM profiles and protein sequences of known SSPs. Then we used the HLH hidden Markov model (HMM) search and TBLASTN search (https://phytozome-next.jgi.doe.gov/blast-search) to make sure the classification of SSP is accurate and complete. For CRP type SSPs prediction, the number of cysteine residues in each SlSSP after removing signal peptide was recorded. The final results were manually revised based on their conserved mature sequences.

### Bioinformatic analysis

The TBtools software (Chen et al. 2020a, b) was used to visualized the chromosomal localization of *SlSSP* genes. The sequence alignment analysis was performed using the DNAMAN software (v 6.0) (Lynnon, Pointe-Claire, QC, Canada).

Two published tomato RNA-seq data of tomato were used to analyze the expression patterns of *SlSSP* genes in various tissues (D004, http://ted.bti.cornell.edu/) and drought stress (GSE151277, https://www.ncbi.nlm.nih.gov/geo/). The GO analysis of *SlSSPs* was performed using the agriGO v2.0 online tool (http://systemsbiology.cau.edu.cn/agriGOv2/index.php) (Tian et al. 2017).

CEP precursor proteins from *Arabidopsis*, *Medicago*, tomato, wheat, rice, *Setaria* and maize were used to construct a phylogenetic tree using MEGA 7 software (Kumar et al. 2016) with the Maximum-likelihood method and 1,000 bootstrap replications. The iTOL online tool (https://itol.embl.de/itol.cgi) (Ciccarelli et al. 2006) was then used to modify the phylogenetic tree. The MEME Suite (https://meme-suite.org/meme/doc/meme.html) (Bailey and Elkan 1994) was used to search for conserved domains and comparative analysis of domain conservation for each CEP protein. The IBS software (Liu et al. 2015) was used to construct the protein structure of CEPs. The promoter sequences (upstream 2 kb sequences) of all *CEP* genes were obtained from NCBI (https://www.ncbi.nlm.nih.gov/) and analyzed using the PlantCARE online tool (https://bioinformatics.psb.ugent.be/webtools/plantcare/html/) (Lescot et al. 2002). The TBtools software (Chen et al. 2020a, b) and an online platform (https://www.bioinformatics.com.cn) were utilized to perform the hierarchical cluster analysis and visualization.

### Identification of unannotated secreted peptides

Firstly, we screened the coding sequence (CDS) and non-coding sequence (NCDS) on all the 12 tomato chromosomes. The small ORFs (sORFs) encoding proteins 25 to 250 amino acids were obtained by translating tomato NCDS in six-frames using the ORF finder package (https://www.ncbi.nlm.nih.gov/orffinder/). Next, the smaller ORFs were eliminated if multiple overlapping in-frame ORFs were recovered. Then, the same procedure used for annotated genes was used to identify the unannotated secreted peptides encoded by sORFs.

### Plant materials and stress treatments

The tomato cultivar Condine Red (referred to as 'CR') was used in this study. Tomato seeds were germinated at 28℃ in darkness for three days and then sown on equal matrix of peat and perlite (3:1; v/v), and put in a growth room. When the first two leaves were fully expanded, tomato plants were transferred to pots containing the equal same matrix and poured permeable. The growth conditions were as follow: light intensity, 200 μmol·m-2·s^−1^; photoperiod, 16 h/8 h; day/night temperature cycle, 26 °C/22 °C; and relative humidity, 70%. 30-day-old plants were used for all the experiments, and drought stress was performed through water withdrawal for 12 d and rehydrated for 6 h. Plants grown under normal growth conditions were used as control.

The tomato plants used for gene expression analysis under drought stress were transferred to hydroponics and grown for 5 d. Then, 20 plants were treated with 20% (m/v) PEG 6000 (BBI, China), 200 nM NaCl or 100 µM ABA (Sigma, America) for 0 h, 3 h, 6 h and 12 h respectively to simulate abiotic stress. Leaves were collected separately and were frozen immediately in liquid nitrogen and stored at -80 °C for further use.

### qRT-PCR

Total RNA from leaves was extracted using an RNAsimple Total RNA Kit (Tiangen Biotech, China) according to the manufacturer’s instructions. 1 µg total RNA was used as the templet to synthesize cDNA using the HiScript®II Q RT SuperMix Kit (Vazyme, China). qPCR was then performed using the Applied Biosystems StepOne™ RealTime PCR System with the ChamQ SYBR qPCR Master Mix Kit (Vazyme, China). The relative transcript level of each gene was calculated using the 2^−ΔΔCT^ method and tomato *Actin* (*Solyc03g078400*) was as the reference gene. All primers for qPCR were designed by the Primer Premier 5.0 software (www.PremierBiosoft.com) and are listed in Supplementary Table S4. All qRT-PCR experiments were run in three biological replicates and three technical replicates.

### Peptide synthesis and treatments

The SlCEP10 (YLGIKNSGPSPGEGH) and SlCEPb (TLGGIKAGPSPGEGH) peptides were chemically synthesized by GenScript Biotech Corporation (Nanjing, China), with a purity ≥ 95% (w/w). Peptides were dissolved in double distilled water (ddH_2_O) at 1 mM as a stock concentration, and then stored at -80 °C for future use. For peptide treatments, distilled water or 5 µM SlCEP peptides were sprayed evenly to tomato leaves at 9:00 am every day, respectively. Each treatment consisted of three biological replicates and each replication consisted of 5 seedlings.

### Morphological and physiological measurements

The 30-day-old tomato plants were photographed with a Canon EOS 80D to obtain high-resolution images. 8 days after treatments, leaves were harvested and weighted immediately after detached from plants.

Relative water content (RWC) was detected on 15 leaves for each treatment. Fresh weight (FW) was recorded and then leaves were placed in distilled water at 4 °C for 24 h to get saturated weight (SW). Finally, leaves were dried at 65 °C for 6 h to determine dry weight (DW). RWC was calculated as the following formula (Terzi and Kadioğlu 2006):

$$\mathrm{RWC}=\left[\left(\mathrm{FW}-\mathrm{DW}\right)/\left(\mathrm{SW}-\mathrm{DW}\right)\right]\times 100\%$$

0.3 ɡ leaf disks (0.6 cm of diameter) from each treatment were harvested for relative electric conductivity (REC) measurements. Samples were placed in a 50 mL tube containing 30 mL distilled water and sharked at 200 rpm at 28 °C for 2 h, then the initial electrical conductivity (K1) was measured with a conductivity meter. After that, the tubes were boiled at 95 °C for 20 min then cooled to room temperature. Finally, the final electrical conductivity (K2) was measured. REC was calculated as the following formula (Cao et al. 2007):

$$\mathrm{REC}=\mathrm{K}1/\mathrm{K}2\times 100\mathrm{\%}$$

The level of MDA was determined using the thiobarbituric acid method (Hodges et al. 1999). 0.3 ɡ leaf sample for each treatment was ground with 3 ml of ice-cold 50 mM PBS, then centrifuged at 12,000 rpm for 20 min at 4 °C. 1 mL supernatant and 3 mL TBA (BBI, China) work solution (20% TCA containing 0.6% thiobarbituric acid) were mixed together and boiled at 95 °C for 30 min. The mixture was immediately cooled on ice to ambient temperature, then centrifuged at 1500 ɡ for 10 min. Absorbance was recorded at 532, and 600 nm using the T6 UV/VIS spectrophotometer (Persee, China). The MDA concentration according to following formula:

$$\mathrm{MDA }\left(\mathrm{nM}/\mathrm{g}\right)=\left[\left(\mathrm{A}532-\mathrm{A}600\right)\times {\mathrm{V}}_{\mathrm{r}}/\upvarepsilon \times {10}^{9}\right]/\left({\mathrm{W}}_{\mathrm{t}}\times {\mathrm{V}}_{\mathrm{t}}/\mathrm{V}\right)$$.

V_r_: Volume of reaction mixture.

V: Total volume of crude enzyme solution.

V_t_: Volume of samples used in the test.

W_t_: Fresh weight of samples used in the test.

Ɛ: Extinction coefficient (1.55 × 10^5^ L/mol/cm).

### Statistical analysis

All experiments adopted a completely randomized design with three biological replicates. The Graph Pad Prism 8 software was used to organize data. Significance of difference was determined via ordinary one-way ANOVA (*p* < 0.05).

### Supplementary Information

**Additional file 1. **The online version contains supplementary materials available at (web address will be provided by the publisher). **Supplementary Fig. S1.** The distribution of sORFs and annotated proteins. **Supplementary Fig. S2.** Chromosome distribution and sequence alignment of *SlCEP* genes. **Supplementary Fig. S3.** Heatmap visualization of the expression pattern of *SlCEPs* under drought (20% PEG-6000) stress for 0, 3, 6 and 12 hour based on qRT-PCR. **Supplementary Fig. S4.** Expression level of *SlSRK2C*, *SlCEP10*, *SlCEP11* under drought (20% PEG6000 for 6 h), salt (200 mM NaCl for 3 h) and ABA (100 μM ABA for 6 h) stress treatments. **Supplementary Fig. S5.** **Supplementary Table S1.** SlSSPs prediction. **Supplementary Table S2.** sORFs prediction (ORF finder). **Supplementary Table S3.** GO analysis of SlSSPs. **Supplementary Table S4.** List of primers used in this study.

## Data Availability

All data are publicly accessible listed under “Gene and Accession Numbers”. Seeds of tomato cultivar Condine Red are commercially available.

## Abbreviations

ABRE: Abscisic acid-responsive elements
ARE: Antioxidant-responsive element
AREB: ABA responsive element binding
CDS: Coding sequence
CEP: C-terminally encoded peptide
CLE: CLAVATA3 (CLV3), CLV3/ESR
CRP: Cys-rich peptide
DRE/MBS: Dehydration-responsive element
DHN: Dehydrin
DW: Dry weight
EPF: Epidermal patterning factor
ERE: Ethylene-responsive element
GLV/RGF/CLEL: Golven/root growth factor
FW: Fresh weight
GO: Gene ontology
GSDS: Gene Structure display Server
HMM: HLH hidden Markov model
IDA: Inflorescence deficient in abscission
K/HDEL: Lys/His-Asp-Glu-Leu
LEA: Late embryogenesis abundant
LTR: Low temperature-responsive element
MtSSPdb: *Medicago**truncatula* Small Secreted Peptide Database
NCDS: Non-coding sequences
NCED: 9-Cis-epoxycarotenoid dioxygenase
ORF: Open reading frame
PEP: Plant elicitor peptides
PIP: (PAMP)-induced peptide
PP2C: Protein phosphatase 2C
PSK: Phytosulfokine
PSY: Plant peptide containing sulfated tyrosine
PTM: Post-translationally modified
RALF: Rapid alkalinization factor
REC: Relative electric conductivity
RLK: LRR-type receptor-like kinase
RWC: Relative water content
SGN: Sol Genomics Network
SRK2C: SNF1-related kinase
SSP: Small secreted peptide
SW: Saturated weight
SYS: Systemin
TC-rich repeat: Defense and stress-responsive element
TM: Transmembrane
