# Supplementary figures and images for: Small secreted peptides (SSPs) in tomato and their potential roles in drought stress response
Source: Mol Hortic. 2023 Aug 25;3:17. doi: 10.1186/s43897-023-00063-2 (PMC10515272; doi:10.1186/s43897-023-00063-2)

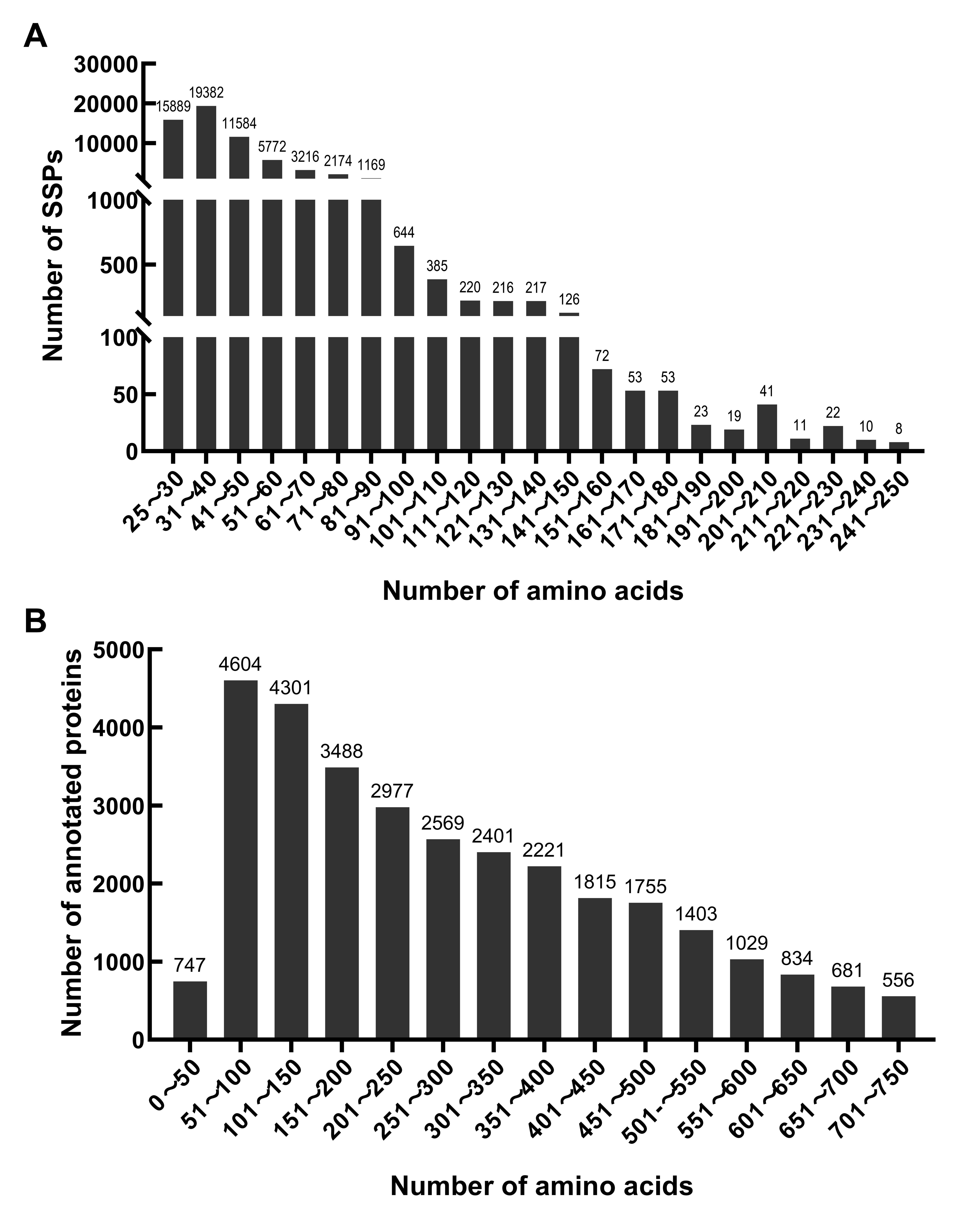

Supplement: Supplementary file 1 — Additional file 1. The online version contains supplementary materials available at (web address will be provided by the publisher). Supplementary Fig. S1. The distribution of sORFs and annotated proteins. Supplementary Fig. S2. Chromosome distribution and sequence alignment of SlCEP genes. Supplementary Fig. S3. Heatmap visualization of the expression pattern of SlCEPs under drought (20% PEG-6000) stress for 0, 3, 6 and 12 hour based on qRT-PCR. Supplementary Fig. S4. Expression level of SlSRK2C, SlCEP10, SlCEP11 under drought (20% PEG6000 for 6 h), salt (200 mM NaCl for 3 h) and ABA (100 μM ABA for 6 h) stress treatments. Supplementary Fig. S5. Supplementary Table S1. SlSSPs prediction. Supplementary Table S2. sORFs prediction (ORF finder). Supplementary Table S3. GO analysis of SlSSPs. Supplementary Table S4. List of primers used in this study. [file 43897_2023_63_MOESM1_ESM.zip › figure S1.jpg]

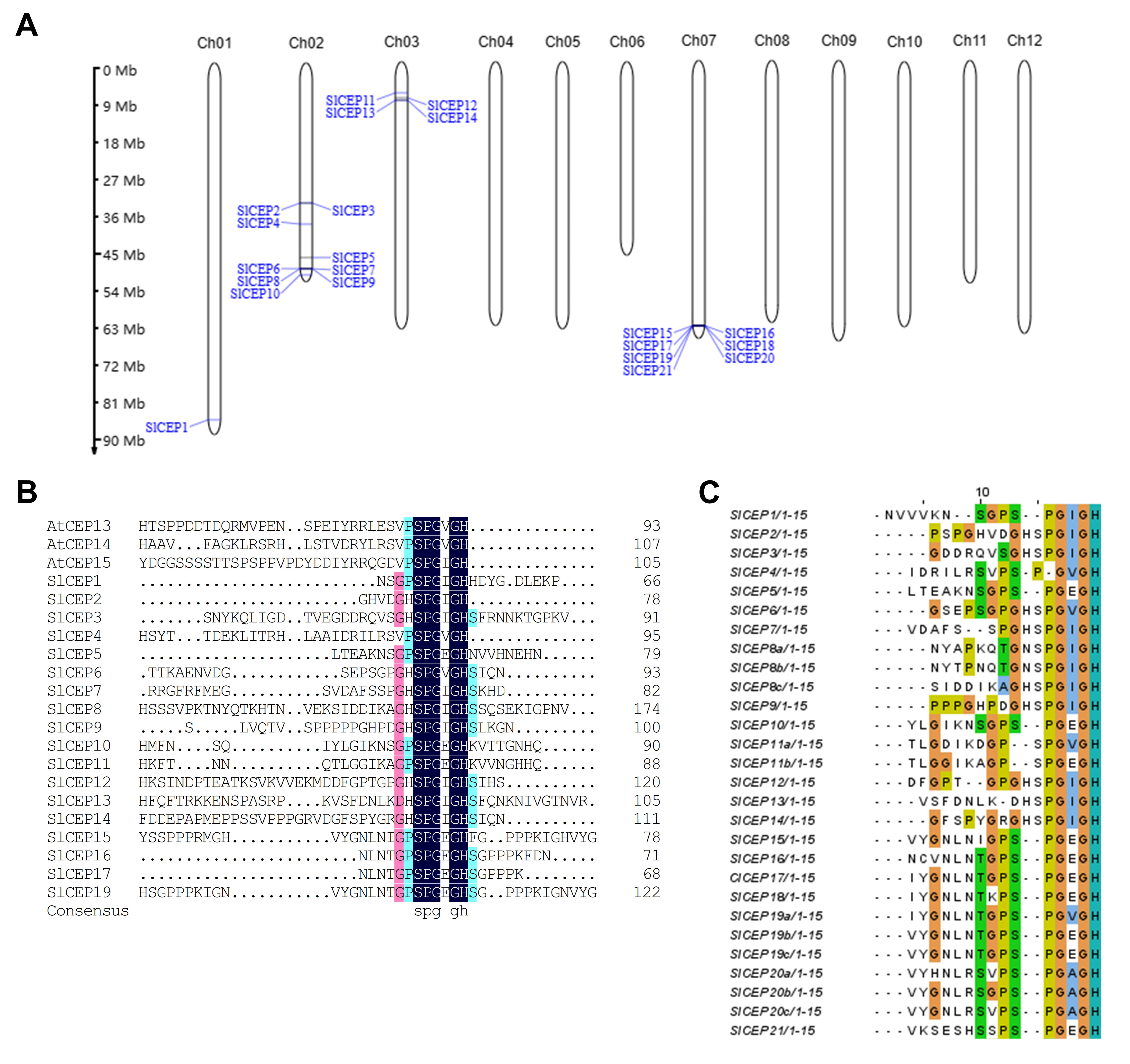

Supplement: Supplementary file 1 — Additional file 1. The online version contains supplementary materials available at (web address will be provided by the publisher). Supplementary Fig. S1. The distribution of sORFs and annotated proteins. Supplementary Fig. S2. Chromosome distribution and sequence alignment of SlCEP genes. Supplementary Fig. S3. Heatmap visualization of the expression pattern of SlCEPs under drought (20% PEG-6000) stress for 0, 3, 6 and 12 hour based on qRT-PCR. Supplementary Fig. S4. Expression level of SlSRK2C, SlCEP10, SlCEP11 under drought (20% PEG6000 for 6 h), salt (200 mM NaCl for 3 h) and ABA (100 μM ABA for 6 h) stress treatments. Supplementary Fig. S5. Supplementary Table S1. SlSSPs prediction. Supplementary Table S2. sORFs prediction (ORF finder). Supplementary Table S3. GO analysis of SlSSPs. Supplementary Table S4. List of primers used in this study. [file 43897_2023_63_MOESM1_ESM.zip › figure S2.jpg]

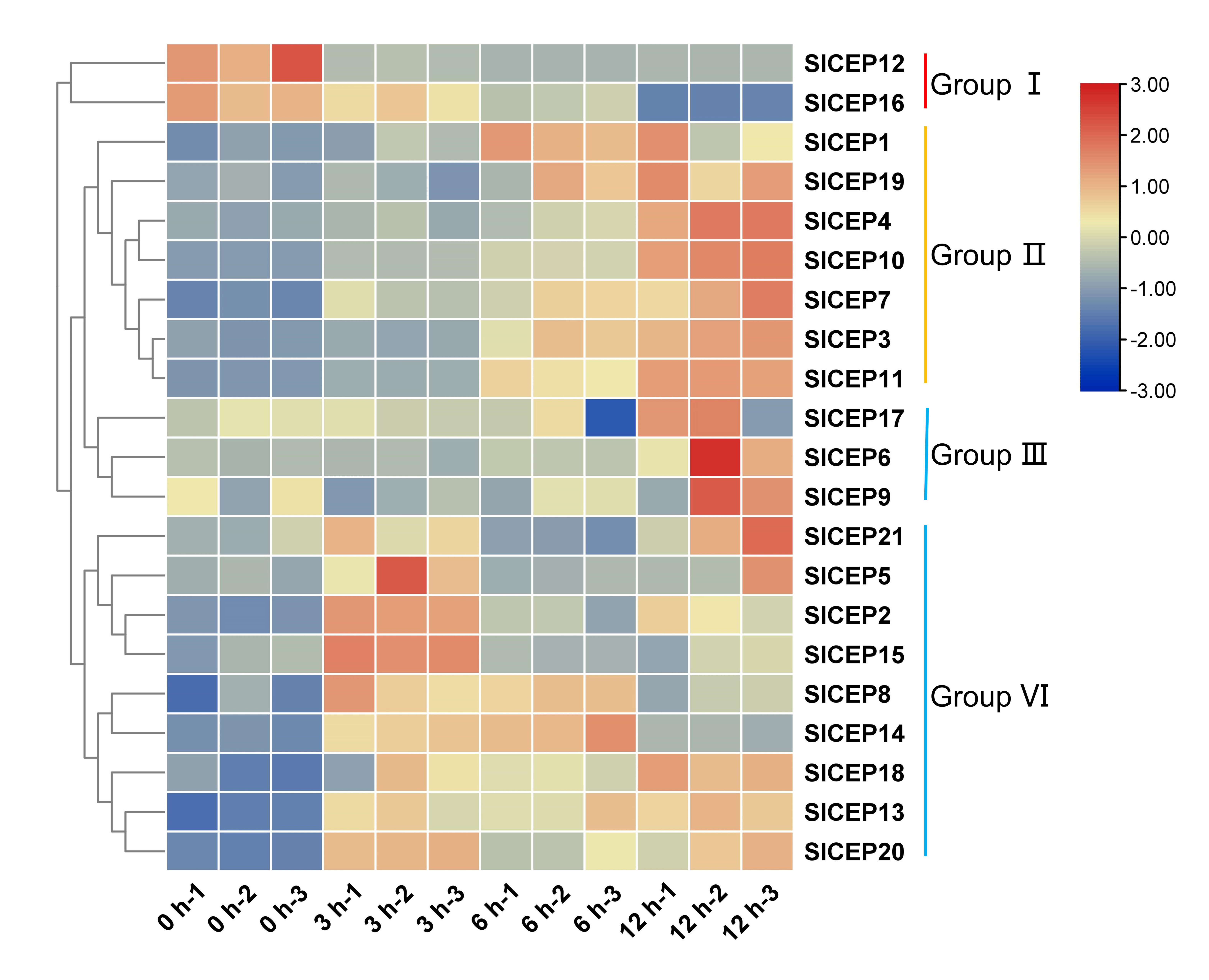

Supplement: Supplementary file 1 — Additional file 1. The online version contains supplementary materials available at (web address will be provided by the publisher). Supplementary Fig. S1. The distribution of sORFs and annotated proteins. Supplementary Fig. S2. Chromosome distribution and sequence alignment of SlCEP genes. Supplementary Fig. S3. Heatmap visualization of the expression pattern of SlCEPs under drought (20% PEG-6000) stress for 0, 3, 6 and 12 hour based on qRT-PCR. Supplementary Fig. S4. Expression level of SlSRK2C, SlCEP10, SlCEP11 under drought (20% PEG6000 for 6 h), salt (200 mM NaCl for 3 h) and ABA (100 μM ABA for 6 h) stress treatments. Supplementary Fig. S5. Supplementary Table S1. SlSSPs prediction. Supplementary Table S2. sORFs prediction (ORF finder). Supplementary Table S3. GO analysis of SlSSPs. Supplementary Table S4. List of primers used in this study. [file 43897_2023_63_MOESM1_ESM.zip › figure S3.jpg]

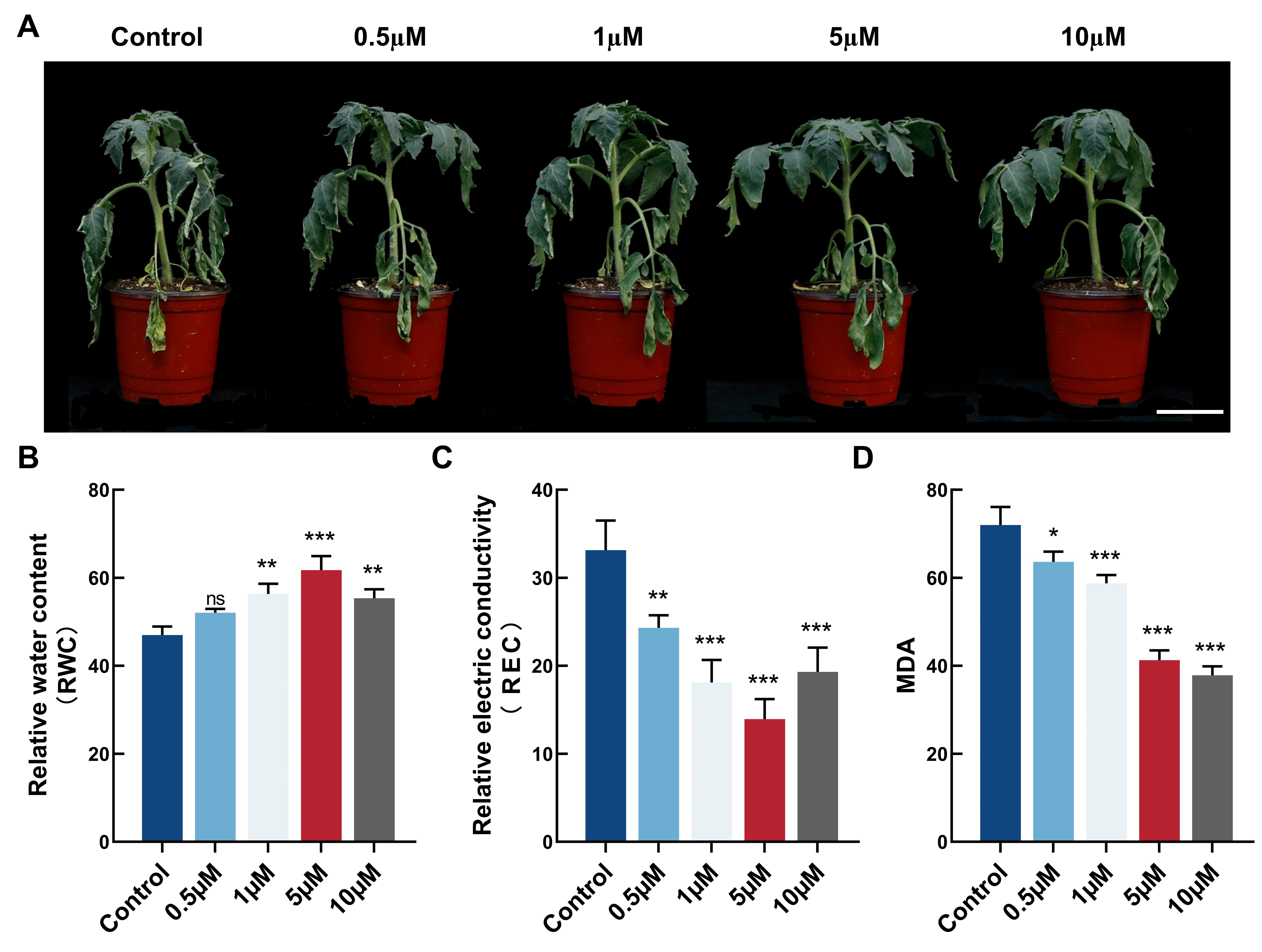

Supplement: Supplementary file 1 — Additional file 1. The online version contains supplementary materials available at (web address will be provided by the publisher). Supplementary Fig. S1. The distribution of sORFs and annotated proteins. Supplementary Fig. S2. Chromosome distribution and sequence alignment of SlCEP genes. Supplementary Fig. S3. Heatmap visualization of the expression pattern of SlCEPs under drought (20% PEG-6000) stress for 0, 3, 6 and 12 hour based on qRT-PCR. Supplementary Fig. S4. Expression level of SlSRK2C, SlCEP10, SlCEP11 under drought (20% PEG6000 for 6 h), salt (200 mM NaCl for 3 h) and ABA (100 μM ABA for 6 h) stress treatments. Supplementary Fig. S5. Supplementary Table S1. SlSSPs prediction. Supplementary Table S2. sORFs prediction (ORF finder). Supplementary Table S3. GO analysis of SlSSPs. Supplementary Table S4. List of primers used in this study. [file 43897_2023_63_MOESM1_ESM.zip › figure S4.jpg]

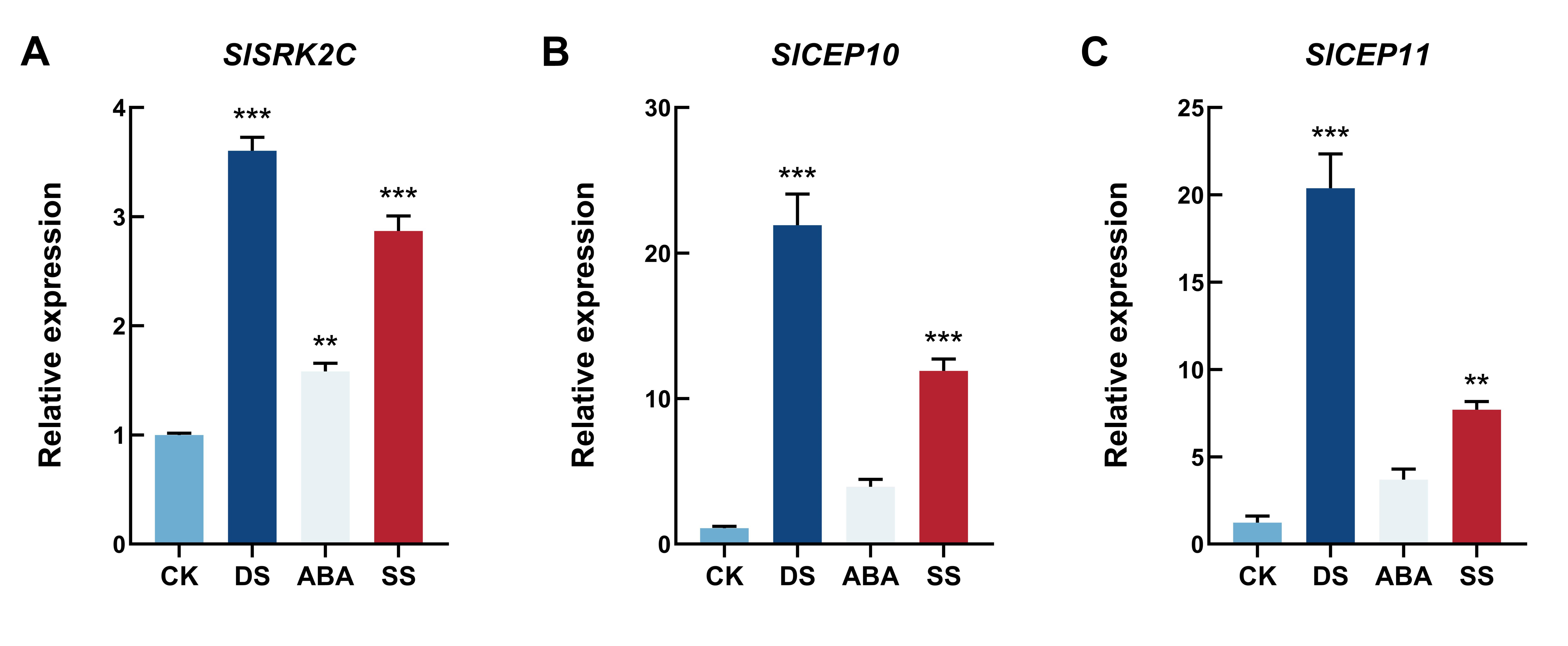

Supplement: Supplementary file 1 — Additional file 1. The online version contains supplementary materials available at (web address will be provided by the publisher). Supplementary Fig. S1. The distribution of sORFs and annotated proteins. Supplementary Fig. S2. Chromosome distribution and sequence alignment of SlCEP genes. Supplementary Fig. S3. Heatmap visualization of the expression pattern of SlCEPs under drought (20% PEG-6000) stress for 0, 3, 6 and 12 hour based on qRT-PCR. Supplementary Fig. S4. Expression level of SlSRK2C, SlCEP10, SlCEP11 under drought (20% PEG6000 for 6 h), salt (200 mM NaCl for 3 h) and ABA (100 μM ABA for 6 h) stress treatments. Supplementary Fig. S5. Supplementary Table S1. SlSSPs prediction. Supplementary Table S2. sORFs prediction (ORF finder). Supplementary Table S3. GO analysis of SlSSPs. Supplementary Table S4. List of primers used in this study. [file 43897_2023_63_MOESM1_ESM.zip › figure S5.jpg]
